# Comprehensive omics analyses profile genesets related with tumor heterogeneity of multifocal glioblastomas and reveal LIF/CCL2 as biomarkers for mesenchymal subtype

**DOI:** 10.7150/thno.65739

**Published:** 2022-01-01

**Authors:** Sheng-Qing Lv, Zhen Fu, Lin Yang, Qing-Rui Li, Jiang Zhu, Qu-Jing Gai, Min Mao, Jiang He, Yan Qin, Xiao-Xue Yao, Xi Lan, Yan-Xia Wang, Hui-Min Lu, Yan Xiang, Zuo-Xin Zhang, Guo-Hao Huang, Wei Yang, Ping Kang, Zhijian Sun, Yu Shi, Xiao-Hong Yao, Xiu-Wu Bian, Yan Wang

**Affiliations:** 1 Department of Neurosurgery, Xinqiao Hospital, Third Military Medical University (Army Medical University), Chongqing, China.; 2 Institute of Pathology and Southwest Cancer Center, Southwest Hospital, Third Military Medical University (Army Medical University), Chongqing, China.; 3 Biobank of Institute of Pathology, Southwest Hospital, Third Military Medical University (Army Medical University), Chongqing, China.; 4 K2 Oncology Co., Ltd, Beijing, China.

**Keywords:** multifocal GBM, molecular subtype, extracellular matrix, immune response, LIF, CCL2

## Abstract

**Rationale:** Around 10%-20% patients with glioblastoma (GBM) are diagnosed with more than one tumor lesions or multifocal GBM (mGBM). However, the understanding on genetic, DNA methylomic, and transcriptomic characteristics of mGBM is still limited.

**Methods:** In this study, we collected nine tumor foci from three mGBM patients followed by whole genome sequencing, whole genome bisulfite sequencing, RNA sequencing, and immunohistochemistry. The data were further examined using public GBM databases and GBM cell line.

**Results:** Analysis on genetic data confirmed common features of GBM, including gain of chr.7 and loss of chr.10, loss of critical tumor suppressors, high frequency of PDGFA and EGFR amplification. Through profiling DNA methylome of individual tumor foci, we found that promoter methylation status of genes involved in detection of chemical stimulus, immune response, and Hippo/YAP1 pathway was significantly changed in mGBM. Although both CNV and promoter methylation alteration were involved in heterogeneity of different tumor foci from same patients, more CNV events than promoter hypomethylation events were shared by different tumor foci, implying CNV were relatively earlier than promoter methylation alteration during evolution of different tumor foci from same mGBM. Moreover, different tumor foci from same mGBM assumed different molecular subtypes and mesenchymal subtype was prevalent in mGBM, which might explain the worse prognosis of mGBM than single GBM. Interestingly, we noticed that LIF and CCL2 was tightly correlated with mesenchymal subtype tumor focus in mGBM and predicted poor survival of GBM patients. Treatment with LIF and CCL2 produced mesenchymal-like transcriptome in GBM cells.

**Conclusions:** Together, our work herein comprehensively profiled multi-omics features of mGBM and emphasized that components of extracellular microenvironment, such as LIF and CCL2, contributed to the evolution and prognosis of tumor foci in mGBM patients.

## Introduction

Glioblastoma (GBM) is the most common primary malignancy in adult brain and 10% - 20% of GBM patients are diagnosed with more than one tumor lesion or multifocal GBM (mGBM) 1, 2. Patients with mGBM have shortened overall survival compared to those with one GBM mass or unifocal GBM (uGBM) and are resistant to current therapeutic measures 3, 4. Previous genetic analysis on mGBM patients reveals the monoclonal nature of different tumor foci from same patients 5, 6, but the genes or genesets involved in tumor evolution of mGBM remain to be elucidated. Given worse prognosis of mGBM than uGBM, identifying genes critically involved in GBM progression might reveal mechanisms underlying dismal prognosis of GBM and provide novel therapeutic targets for both mGBM and uGBM.

According to the transcriptomic profiles and genetic alterations, GBM is classified into three molecular subtypes - proneural, classical, and mesenchymal with distinct genetic and transcriptomic features, respectively 7, 8. Proneural subtype is significantly associated with copy number amplification of PDGFRA gene and point mutations in IDH1, and showed high expression of PDGFRA, NKX2-2, and OLIG2, but reduced expression of CDKN1A tumor suppressor gene. Gene ontology (GO) categories of proneural transcriptome include developmental processes and cell cycle/proliferation signature 9. Classical subtype is featured with EGFR gene amplification and homozygous deletion of Ink4a/ARF locus. In addition, Notch and Sonic hedgehog signaling pathways are highly expressed in this subtype 7. Mesenchymal subtype is characterized by high expression of CHI3L1 and MET, high frequency of NF1 mutation/deletion, and low levels of NF1 mRNA expression. Signature genes of wound healing and NF-κB signaling pathway is remarkably involved in mesenchymal subtype. In clinical prognosis and treatment, the three subtypes are corresponding to different survival length and treatment response 7, 8. The existence of different GBM subtypes raises questions regarding their natural history and the sequential events in which individual alterations are incorporated 10, 11. Interestingly, different subtypes also share some common genetic alterations, e.g., gain of chromosome 7 (chr.7) and loss of chr.10 10, 12, implying that various molecular might arise from a common origin 12. Recently, DNA methylation profiling has become an important adjunct tool for tumor classification and identification of molecular subclasses 13-15. Whole genome methylation, especially methylation alterations in promoter area, pivotally regulates gene expression. Genome-wide methylation profiles not only define subgroup of gliomas 13, 16, but also are associated with survival time in GBM patients 15, 17. By now, it has been known that whole-genome methylation, especially promoter CpG methylation, are tightly correlated with glioma phenotype 13-15.

So far, comprehensive omics data, including genomic, methylomic, and transcriptomic profiles of GBM have been extensively investigated, which delineate novel disease-driver mutations and chromosomal rearrangement events and provide new molecular subtype classification according to genetic alterations, methylation, and transcription 7, 8, 13, 18-20. Moreover, IDH1 mutation has been found to act as oncogenic events through comprehensively modifying metabolism and methylation profiles to drive glioma development 21-24. As a specific pathological population of GBM, mGBM remains to be investigated through comprehensive omics. Abou-El-Ardat *et al.* has reported the genomic characteristics of eleven tumor foci from five mGBM cases without profiling transcriptome and methylome 6. Liu *et al.* has reported the comparison of genetic characteristics of mGBM with uGBM using TCGA database but no omics information of individual tumor foci from mGBM 25. By now, investigation on transcriptomes and methylomes on different tumor foci of mGBM has not yet been performed and it is unclear which genes/signaling pathways are pivotally involved in the progression of different tumor foci of mGBM. Therefore, in this work, we comprehensively analyzed multi-omics of nine tumor foci from three mGBM patients and revealed several genes/genesets tightly related with tumor evolution of mGBM.

## Results

### Different tumor foci from same mGBM patient shared critical genetic alterations of GBM

**Figure 1:**
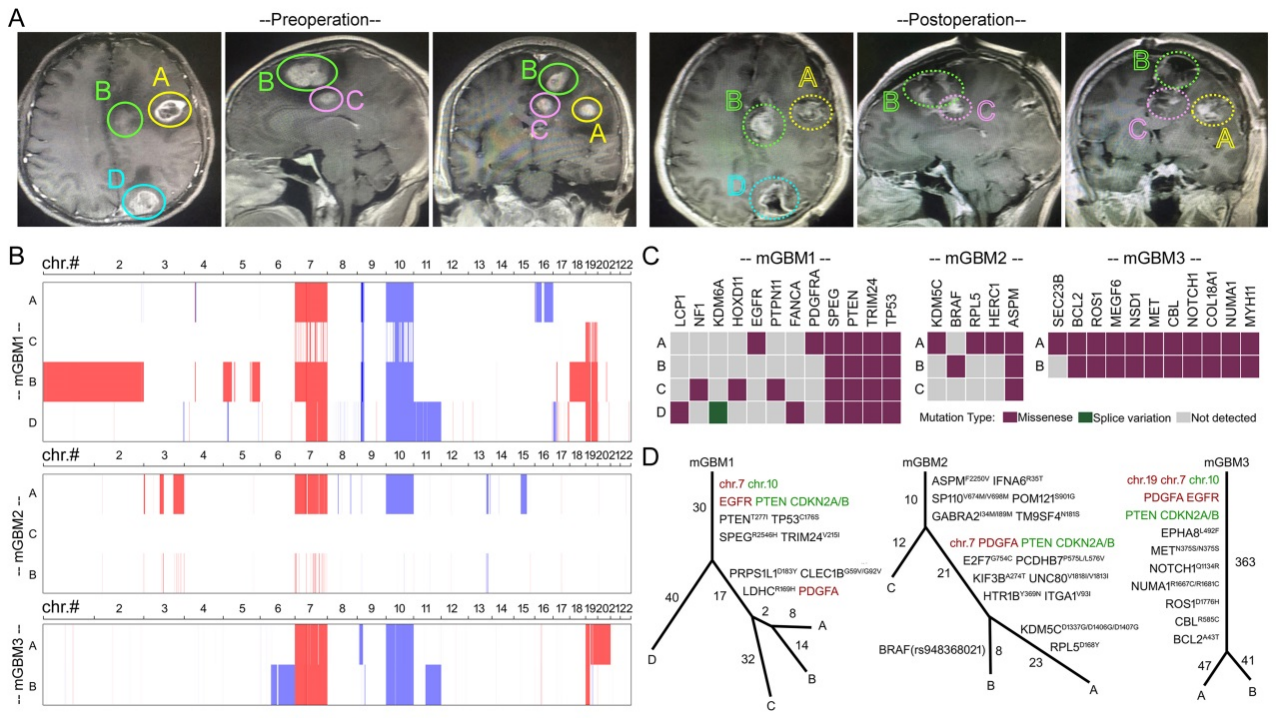
** Analysis on genomes of tumor foci from the three mGBM patients.** A) Representative MRI images of mGBM1 patient. Solid line circle in upper panels and dash line circle in lower panels indicate the tumor areas before and after surgical removal, respectively. B) Diagram of chromosomal copy number variation of tumor foci from the three mGBM patients. Red color and blue color represent chromosomal gain and loss, respectively. C) Distribution of cancer driver genes with mutations in tumor foci from the three mGBM patients. D) Evolution route of tumor foci from the three mGBM patients according to CNV and SNV. Red color font and green color font represent chromosomal gain and loss, respectively.

Here, two male patients (47 and 72 years old, respectively) and one female patient (36 years old) **(Table S1)** were subjected to neuro-navigation and fluorescein-guided surgery **(Figure 1A, Figure S1A, and S1B)** in Xinqiao hospital, Chongqing, China. We collected totally nine tumor foci from three mGBM patients (mGBM1, mGBM2, and mGBM3). mGBM1, mGBM2, and mGBM3 included four tumor foci (mGBM1_A-D), three tumor foci (mGBM2_A-C), and two tumor foci (mGBM3_A and mGBM3_B), respectively. In our cases, all tumor foci localized in the same cerebral hemisphere, which was consistent with previous reports, in which most of patients (5 of 6 cases 6 and 30 of 35 cases 25) developed tumor foci in the same cerebral hemisphere. HE staining confirmed that all tumor foci assumed pathological features of GBM **(Figure S1C)**. Then, all tumor foci were subjected to whole-genome sequencing (WGS) for genome, whole genome bisulfite sequencing (WGBS) for methylome, and RNA sequencing (RNA-Seq) for transcriptome followed by bioinformatic analyses.

We first profiled the genetic features of nine tumor foci from three mGBM patients through WGS. The data showed that all tumor foci lacked of IDH1/2 mutations **(Data not shown)** and tumor foci of mGBM1 and mGBM2 carried TERT promoter mutation C228T (chr.5:1,295,228:C>T) 26 **(Table S2)**. As reported previously 6, 10, 12, 25, gain of chr.7 and loss of chr.10 were identified as common features for GBM (mGBM and uGBM) **(Figure 1B) (Table S3)**. In addition, loss of chr.9 and gain of chr.19 were also detected as frequent events in our study **(Figure 1B) (Dataset 1)** and previous reports 6, 25. Moreover, in the tumor foci, we detected alterations of signature genes broadly reported in GBM. Losses of PTEN and CDKN2A/CDKN2B 7, 20 were frequent events in 7 and 8 tumor foci, respectively **(Table S3)**. Amplification of PDGFA gene and EGFR gene was detected in 7 tumor foci **(Table S3)**. It should be mentioned that PDGFA was a prognostic marker for glioma and analyses on several glioma databases consistently indicated that high expression of PDGFA predicted poor survival **(Figure S1D)**. Our study observed high frequency of PDGFA gene amplification with 3 in 3 patients (100%) and 7 in 9 tumor foci (77.8%), which was comparable to previous report by Abou-El-Ardat *et al*. with 6 in 6 patients (100%) and 9 in 12 tumor foci (75%) 6.

Since derivation of multiple tumor foci is important for understanding the progression of mGBM, we explored tumor evolution routes of the three mGBM patients according to copy number variations **(Dataset 1)** and single nucleotide variations **(Dataset 2)**. We found that different tumor foci from same patients shared critical driver mutations** (Figure 1C)**. The evolution simulation indicated that mGBM1_D and mGBM1_A, B, C took different evolution route due to variations of mutation accumulation, but mGBM1_A and B were close to each other at the mutation levels **(Figure 1D)**. For mGBM2, the simulation of evolution route implied that mGBM2_C and the other two foci might develop separately in very early stage, but mGBM2_B and A were close to each other in evolution route **(Figure 1D)**. For mGBM3, the two foci shared most significant mutations **(Figure 1D)**, supporting their common origination. Together, these data revealed common features of GBM, including gain of chr.7 and loss of chr.10, lacking IDH1/2 mutations, high frequency of TERT promoter mutation, loss of critical tumor suppressors (e.g., PTEN and CDKN2A/CDKN2B), high frequency of PDGFA and EGFR amplification. Critical oncogenic alterations in genome were shared by different tumor foci of mGBM, emphasizing the pivotal roles of genetic events in mGBM initiation.

### Methylomic analysis profiled common features and genesets in tumor foci of mGBM

In GBM, CpG methylation profiles play critical roles for classification of molecular subtypes 13, 20, 27, but methylomic features of individual tumor focus of mGBM have not yet been studied. Thus, we performed WGBS to profile DNA methylation features of the nine tumor foci **(Dataset 3)**. A specific G-CIMP has been identified in glioma, featured with somatic IDH1/2 mutation, lack of chr.7 amplification, obvious amplification of 8q23.1-24.3 and 10p15.3-p11.21, and prevalence in low-grade glioma but not GBM 13, 20. All samples in our study were confirmed as GBM, harbored wild-type IDH1/2, and had no typical chromosomal alterations of G-CIMP, indicating Non G-CIMP characteristics of our samples. Principle component analysis (PCA) showed that genomic methylation of different tumor foci from same patients were remarkably varied **(Figure S2A)**. Methylation pattern and gene expression correlation showed that in all samples, CG methylation account for the major methylation events and high CG methylation remarkably leaded to gene silence **(Figure S2B)**. Methylomic heatmap cluster and PCA on the nine tumor foci revealed that despite significant diversity of methylomes of the nine tumor foci, tumor foci from same patients could be preferentially clustered together with an exception of mGBM1_A **(Figure S2A and S2C)**.

**Figure 2:**
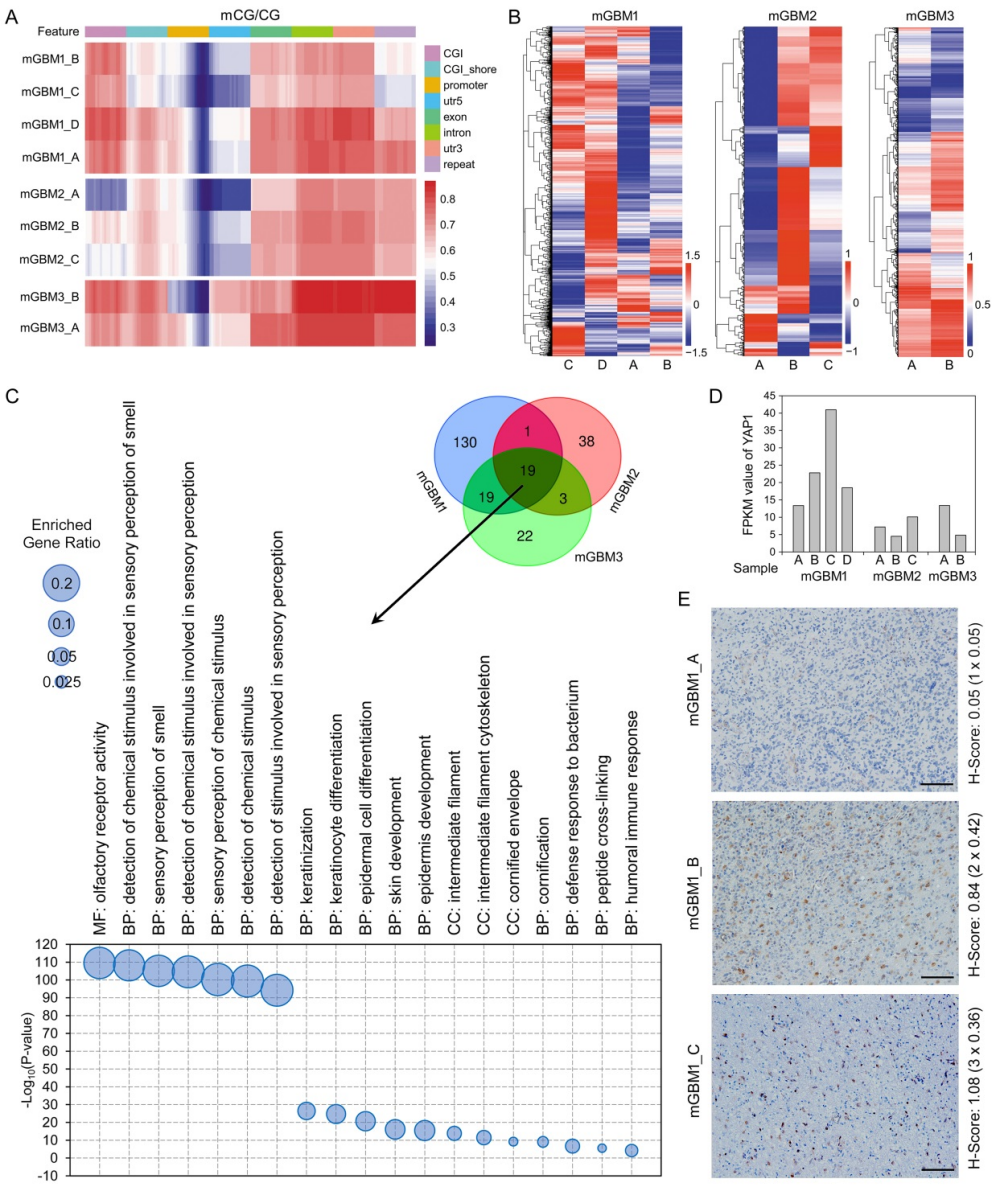
** Analysis on genetic methylomes of tumor foci from the three mGBM patients.** A) Heatmap diagram showing the comparison of methylated CpG with total CpG in different gene regions of tumor foci from the three mGBM patients. Red color and blue color represent hypermethylation and hypomethylation, respectively. B) Heatmap cluster of methylomes of tumor foci from the three mGBM patients. C) Venn diagram and scatter graph showing 19 GO genesets consistently enriched by genes with altered promoter CpG methylation in all three mGBM patients. D) FPKM value of YAP1 in tumor foci from the three mGBM patients. E) IHC of YAP1 in three tumor foci from mGBM1. H-Score (intensity × percentage) for each slide were labelled at the right of images. Scale bars: 100 μm.

Although methylation levels in different chromosomes of tumor foci from same patients were varied, chr.22, 17, and 16 were the most frequently hypomethylated chromosomes in the three mGBM patients **(Table S4)**. Then, we measured the methylation level in different DNA regions and found that the promoter region harbored the lowest methylation level among all measured regions **(Figure 2A)**. Further analysis showed that the lowest methylation level in promoter was at the location close to the gene body **(Figure S2D)**. Cluster analysis revealed distinguished promoter methylation profiles in different tumor foci from same patients **(Figure 2B)**. To categorize genes with methylation alterations in promoter region, we performed GO analysis and found that 169, 61, and 63 genesets were enriched in mGBM1, 2, and 3, respectively **(Table S5)**. Among these genesets, 19 genesets were consistently enriched in the three patients, including detection of chemical stimulus, epidermal cell development and differentiation, immune response, and intermediate filament **(Figure 2C)**. To further delineate the signaling pathways potentially affected in mGBM evolution, we performed KEGG analysis between tumors from same patients **(Table S6)**. Interestingly, Hippo pathway was consistently enriched in all samples. We then examined the key transcription factor of Hippo pathways, YAP1, in transcriptomes **(Dataset 4)**. In patient 1, mGBM1_C showed the highest YAP1 expression **(Figure 2D)**. IHC of YAP1 protein further confirmed higher nucleus expression of YAP1 in mGBM1_C than mGBM1_A and B **(Figure 2E)**. It was noted that mGBM1_C had the longest evolution route in patient 1. Using TCGA_GBM database, YAP1 mRNA level in mesenchymal subtype was higher than that in proneural and classical subtypes **(Figure S2E)**. Moreover, YAP1 expression consistently predicted worse prognosis in several glioma databases **(Figure S2F-J)**. These data implied that Hippo/YAP1 pathway was critical for mGBM evolution. Together, our results for the first time profiled the chromosomes, DNA regions, and genes susceptible to DNA methylation alterations during mGBM evolution.

### Transcriptomic analysis revealed critical genesets in tumor foci of mGBM

**Figure 3:**
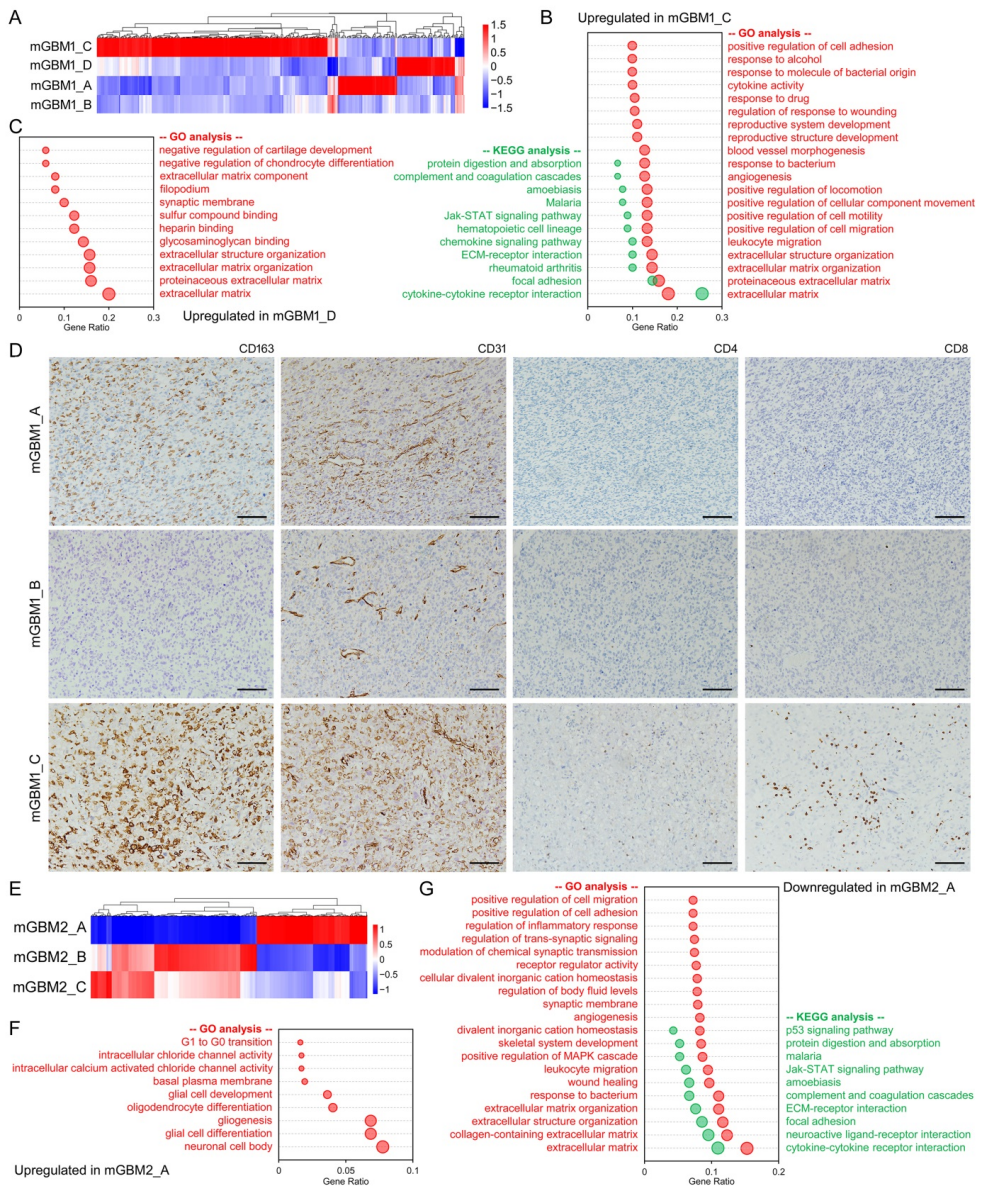
** Analysis on transcriptomes of tumor foci from mGBM1 and mGBM2.** A) Heatmap diagram showing the cluster of gene expression in different tumor foci from mGBM1. B) Gene enrichment of significantly changed mRNA in mGBM1_C compared with the other three tumor foci in mGBM1. C) Gene enrichment of significantly changed mRNA in mGBM1_D compared with the other three tumor foci in mGBM1. D) Representative IHC images of indicated protein markers. Scale bars: 100 μm. E) Heatmap diagram showing the cluster of gene expression in different tumor foci from mGBM2. F) Gene enrichment of significantly changed mRNA in mGBM1_A compared with the other two tumor foci in mGBM2. G) Gene enrichment of significantly changed mRNA in mGBM1_A compared with the other two tumor foci in mGBM2.

We collected RNA-seq data of all tumor foci and analyzed their transcriptomes **(Dataset 4)**. To inspect the purity of the tumor foci, we used ESTIMATE algorithm to analyze mRNA expression data of the nine tumor foci and noticed that the tumor purity value of all tumor foci was above 0.7 **(Table S7)**, which is the most accurate cutoff value to measure tumor purity according to previous report 28. PCA indicated that different tumor foci from same mGBM patient and different mGBM patients were transcriptionally varied **(Figure S3A)**. Cluster analysis revealed dramatically upregulated gene clusters in mGBM1_C compared with the other three mGBM1 tumor foci **(Figure 3A)**. GO analysis and KEGG analysis on these genes indicated that extracellular matrix (ECM), leukocyte migration, angiogenesis, and immune response-related genesets (e.g., cytokine-cytokine receptor interaction, chemokine signaling pathway, JAK-STAT signaling pathway) were significantly enriched **(Figure 3B and Table S8)**. ECM-related genesets were also enriched in upregulated genes in mGBM1_D **(Figure 3C and Table S8)**.

To confirm the results of enrichment in mGBM1, we performed immunohistochemistry (IHC) on mGBM1_A, B, and C using anti-CD31 (for angiogenesis), anti-CD163 (for Macrophage), anti-CD4 (for CD4^+^ T cell), and anti-CD8 (for CD8^+^ T cell). Consistently, IHC data indicated that all tested markers were much higher in mGBM1_C than the other two samples **(Figure 3D)**. In mGBM2, two clusters were established according to gene expression profile of mGBM2_A **(Figure 3E)**. GO and KEGG analyses showed that the upregulated genes in mGBM2_A enriched genesets on neuron, glia, and G1 to G0 transition **(Figure 3F and Table S8)**, but downregulated genes enriched genesets related with ECM, cell migration, immune response, synapse, and angiogenesis **(Figure 3G and Table S8)**. For mGBM3, the upregulated genes in mGBM3_A enriched genesets on ECM, cell migration, transcription regulation, cell cycle regulation, angiogenesis, cytokine-cytokine receptor interaction and signaling pathways in cancers **(Figure S3B and Table S8)**, but the upregulated genes in mGBM3_B enriched genesets on interactions with neurons, synapses, and ion channels **(Figure S3B and Table S8)**, pointing out that mGBM3_A might be more malignant than mGBM3_B. Combination analysis on top 100 enriched GO genesets in all three patients revealed that ECM, migration and adhesion, circulatory system, and synapse proteins were frequently enriched **(Figure S3C)**. Together, these data indicated that tumor microenvironment including ECM, immune response, angiogenesis, and interaction with neuron were frequently altered and thus might be critically involved in mGBM progression.

### Multi-omics analyses indicated intratumor and intertumor heterogeneity of mGBM

**Figure 4:**
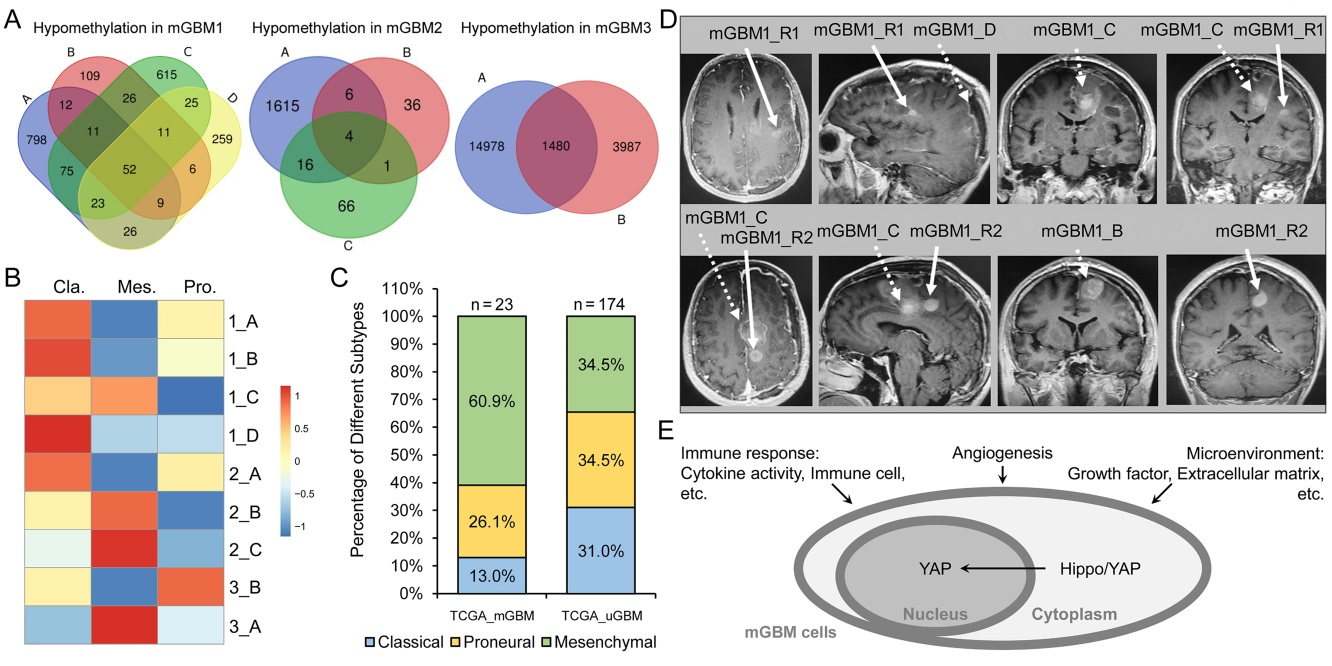
** Molecular subtype classification of tumor foci from the three mGBM patients.** A) Venn diagram of counts of genes with promoter hypomethylation in individual tumor foci of three mGBM samples. B) Molecular subtype of individual tumor focus. C) Percentage of different molecular subtypes of GBM in mGBM and uGBM, respectively. D) The MRI images showing comparison of original tumor foci (mGBM1_B-D) with newly developed tumor foci (mGBM1_R1 and R2) after surgery and standard radio- and chemo-therapy. E) Schematic diagram of commonly altered genesets in our mGBM samples, mGBM *vs.* uGBM (TCGA_GBM), and mesenchymal *vs.* non-mesenchymal (TCGA_GBM).

We noticed that the three tumor foci from mGBM2 harbored fewer CNV events than those from mGBM1 and mGBM3 **(Figure S4A and Dataset 1)**. Consistently, total CNV events of mGBM2 was also fewer than mGBM1 and mGBM3 **(Figure S4A and Dataset 1)**. Therefore, intratumor (tumors from same patient) heterogenicity was in accordance with intertumor (tumors from different patient) heterogenicity. In addition, genes with CNV could be shared by different tumor foci from same mGBM patients, but one or two tumor foci showed significantly specific CNV events **(Figure S4B)**. For example, all gains in mGBM1_C were shared by mGBM1_A, B, and D. mGBM1_B showed the most focus-specific gains but mGBM1_A and D showed significant loss. mGBM2_A harbored the most gain and loss among three tumor foci from mGBM2. CNV in mGBM2_B and C were almost detected in mGBM2_A. In mGBM3, focus A and B showed specific gain and loss, respectively. The shared CNV in different foci from same patients indicated common origin of different foci in early stage of tumor initiation. But with evolution, different tumor foci continued to accumulate tumor-specific CNV, which were importantly involved in tumor heterogeneity. We also analyzed promoter methylation and found that genes with promoter hypomethylation in one sample compared to the other samples from same patients were few overlayed with each other **(Figure 4A and Dataset 3)**. The percentage of focus-specific promoter-hypomethylated genes to full promoter-hypomethylated genes in tumor focus was 79.3% (mGBM1_A), 46.2% (mGBM1_B), 73.4% (mGBM1_B), 63% (mGBM1_B), 98.4% (mGBM2_A), 57.1% (mGBM2_B), 75.9% (mGBM2_C), 91% (mGBM3_A), and 72.9% (mGBM3_B), respectively. Interestingly, although promoter methylation is critical regulation mechanism on gene expression, only less than 20% genes with promoter hypomethylation finally showed increased mRNA levels **(Table S9)**. Thus, a large number of genes with CNV shared by two or more tumor foci from same patients, even though some samples showed unique CNV. For promoter hypomethylation, however, most changed events were focus-specific in same patients. These data implied that genetic changes and methylation status alterations might not be synchronize during tumor evolution.

We then explored the molecular subtype for each tumor focus according to previously reported algorithm by Wang et al 8. Transcriptomic analysis showed that mGBM1_A, B, and D were classical subtype, but mGBM1_C was mesenchymal subtype **(Figure 4B)**. Interestingly, mGBM1_A, B, and D harbored high amplification of EGFR gene **(Table S3)**, which supported their classical subtype classification 7. The mesenchymal feature of mGBM1_C was consistent with the previous results that tumor microenvironment critically contributes to the development of mesenchymal subtype in GBM 8, 29. In addition, critical mesenchymal transcriptional factors were significantly higher in mGBM1_C, than the other samples **(Figure S4C and Dataset 4)**. Hedgehog signaling pathway was enhanced as expected in mesenchymal subtype **(Figure S4C and Dataset 4)**. In mGBM2, both mGBM2_B and mGBM2_C were mesenchymal subtype **(Figure 4B)**, but mGBM2_A was classical subtype with highest EGFR amplification in patient 2 **(Figure 4B and Table S3)**. In addition, downregulated genes in mGBM2_A compared to mGBM2_B and C enriched genesets on ECM, angiogenesis, and immune response, further confirming the contribution of microenvironment to mesenchymal subtype in GBM. In patient 3, mGBM3_A and mGBM3_B were mesenchymal and proneural, respectively **(Figure 4B)**. Analysis on TCGA GBM cases with MRI documents described by Liu *et al.* 25 showed that mesenchymal subtype was more prevalent in mGBM than uGBM (14 in 30 mGBM cases (46.7%) *versus* 60 in 211 uGBM (28.4%) **(Figure 4C)**. The study by Abou-El-Ardat *et al.* indicated that mesenchymal subtype existed in all mGBM cases from their cohort 6. It has been thought that mesenchymal subtype of GBM bears stronger recurrence potential than the other subtypes 30-32. In patient 1, we documented the post-operation data and noticed that there were two recurrent tumor foci (mGBM1_R1 and R2) by 35 days after surgery. It was noted that both R1 and R2 were very close to mGBM1_C, which was identified as mesenchymal subtype in mGBM1 **(Figure 4D)**. By 165 days after surgery, two more new lesions were detected with mGBM1_R1 as the largest one **(Figure S4D)**. Thus, mesenchymal subtype was a major molecular subtype for mGBM and the mesenchymal tumor focus might be critically contributed to the relapse of mGBM.

Besides previously reported core pathways for mGBM 6, including TP53, Receptor Tyrosine Kinases, CDKN2A/2B, and PI3K-AKT **(Table S3 and Dataset 1)**, we also explored genesets correlated with mGBM through combination of analysis on our mGBM samples (CNV, promoter methylation, and transcriptome) and analysis on TCGA database, which have not yet been evaluated in previous mGBM-related work. Our data showed that genesets on immune response, cell-extracellular matrix interaction, and angiogenesis were enriched in our mGBM samples as well as mGBM *vs.* uGBM and mesenchymal *vs.* non-mesenchymal **(Figure 4E and Table S10)**. In addition, Hippo/YAP signaling pathway was also enriched by our mGBM samples, mGBM *vs.* uGBM, and mesenchymal *vs.* non-mesenchymal **(Figure 4E and Table S10)**. Together, mGBM might frequently involve mesenchymal subtype, as well as, increased immune response, interaction between tumor and microenvironment, angiogenesis, and Hippo/YAP signaling pathway.

### LIF and CCL2 were highly expressed by mesenchymal tumor focus in mGBM

**Figure 5:**
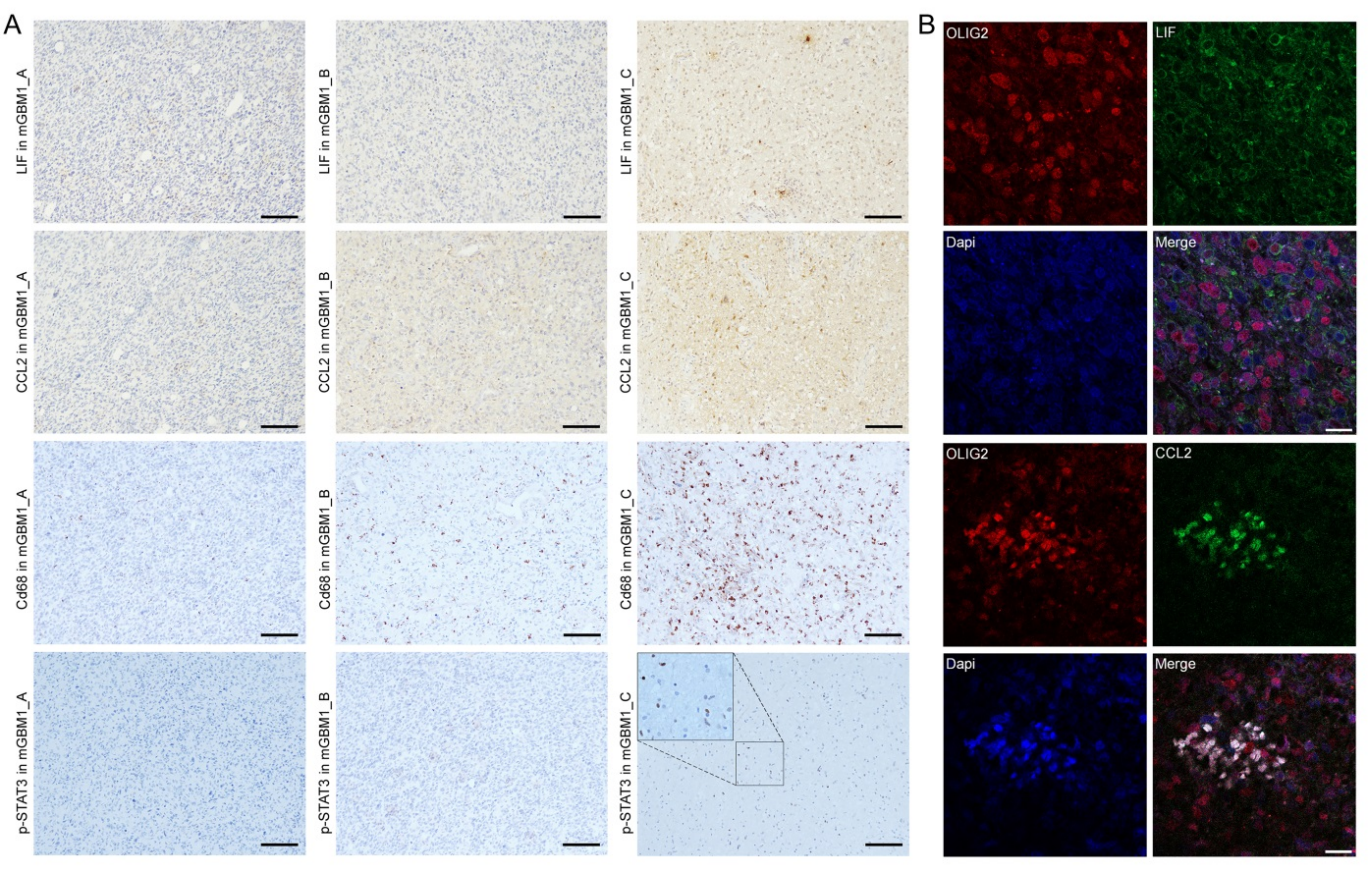
** Expression and clinical significance of LIF and CCL2 in GBM.** A) Representative IHC images of indicated proteins on mGBM1_A-C. For p-STAT3 on mGBM1_C, there is a 2× enlargement (small rectangle to large rectangle) to show the nuclear localization of brown p-STAT3 signal. Scale bars: 100 μm. B) Representative Immunofluorescence images of indicated proteins on mGBM1_C. Dapi is used to show cell nuclei. Scale bars: 20 μm.

Our current study and previous mGBM studies showed that mGBM were significantly associated with the mesenchymal subtype 6, 25, revealing mesenchymal subtype a typical feature of mGBM. It has been known that immune microenvironment is responsible for development of mesenchymal subtype in GBM 8, 29 and results in treatment resistance 32, 33. In our study, both transcriptomic and methylomic analyses indicated the involvement of immune response-related genes in the evolution of tumor foci of mGBM, and it is well-known that cytokines play critical roles for immune response. Thus, we investigated the expression characteristics of cytokines in mesenchymal subtype tumor foci *vs.* non-mesenchymal subtype ones from same mGBM patients. For this purpose, we had got the list of 187 cytokines from Uniprot database using cytokine as keyword **(Table S11)** and analyzed the list in transcriptomes of tumor foci from same mGBM patients. We used 5-fold upregulation as threshold to select interesting cytokines. Intriguingly, the mesenchymal tumor foci in the three mGBM patients consistently highly expressed two cytokines, i.e., LIF and CCL2 **(Figure S5A and Table S12)**. IHC result further confirmed high expression of LIF and CCL2 in mGBM1_C compared to mGBM1_A and B **(Figure 5A)**. Since CCL2 plays strong chemotaxis function to recruit macrophage and LIF activates STAT3 pathway 34-40, we performed IHC to examine macrophage infiltration using CD86 antibody and activation of STAT3 using p-STAT3 antibody. The data indicated the consistence between CCL2 and CD68, as well as, LIF and p-STAT3 **(Figure 5A)**.

To investigate the cell source of the two cytokines, we checked literature and analyzed data of the single cell sequencing on GBM by Wang *et al.* 41. CCL2 was found expressed by GBM cells and macrophages 34-37, but LIF mainly expressed in GBM cells 38-40, which were also supported by single cell sequencing data **(Figure S5B)**. Through immunofluorescence, we found that LIF rarely expressed in macrophages labelled with CD68 **(Figure S5C)**, but highly expressed in GBM cells labelled with Olig2 **(Figure 5B)**. However, CCL2 was localized in both GBM cells and macrophages **(Figure 5B and S5C)**.

### LIF and CCL2 were tightly correlated with poor prognosis of GBM patients

**Figure 6:**
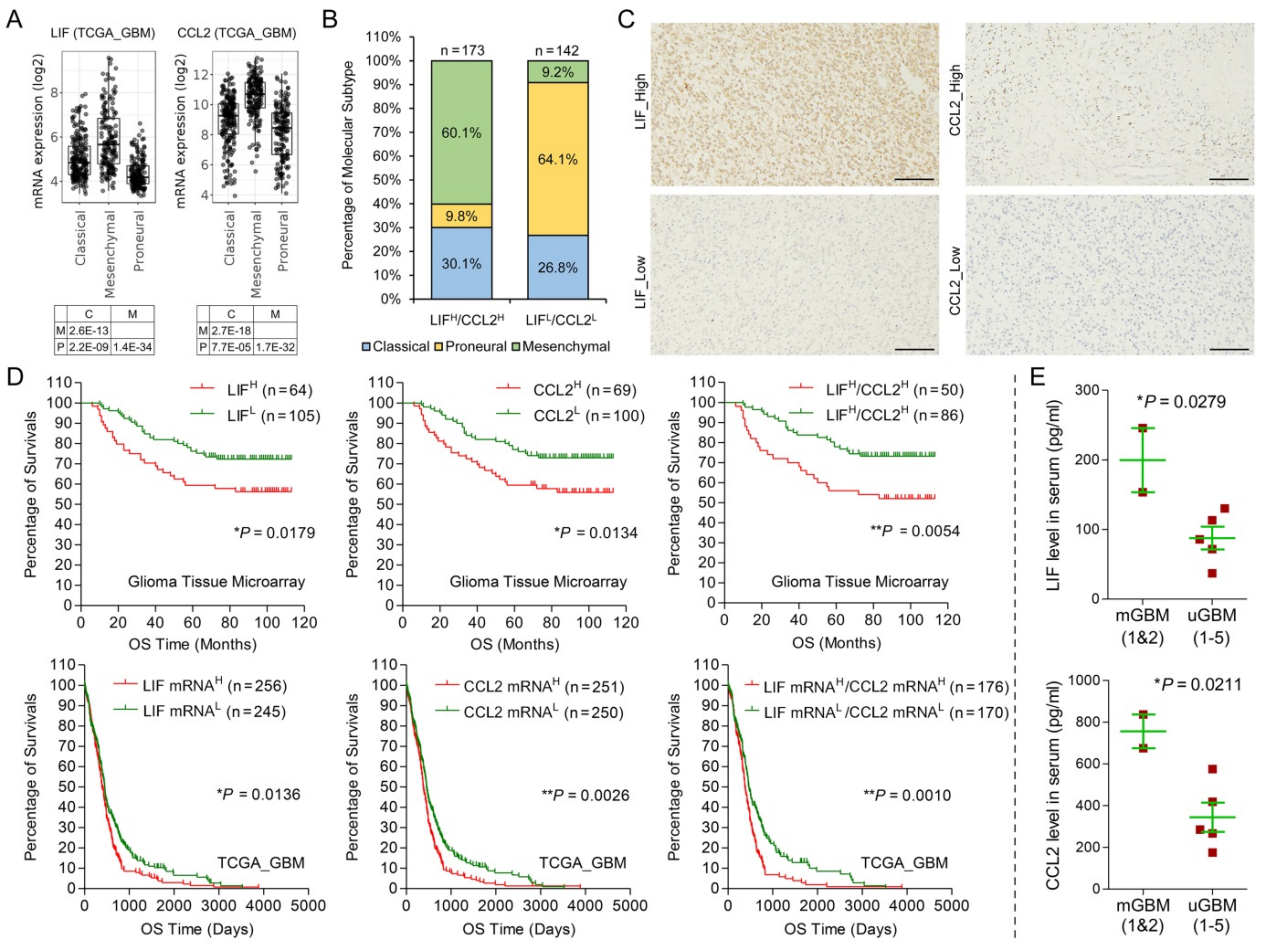
** Clinical significance of LIF and CCL2 in GBM.** A) mRNA expression of LIF and CCL2 in different molecular subtypes using TCGA_GBM database. B) Percentage of different molecular subtypes of GBM with LIF^High^/CCL2^High^ and LIF^Low^/CCL2^Low^, respectively. C) Representative IHC images of indicated proteins on glioma tissue microarray. Scale bars: 100 μm. D) Kaplan-Meier survival analysis on protein levels of LIF or/and CCL2 in our glioma tissue microarray and mRNA levels of LIF or/and CCL2 in TCGA_GBM database. E) Measurement on protein levels of LIF and CCL2 in serum from 2 mGBM patients (mGBM1 and mGBM2) and additional 5 uGBM patients through ELISA.

Then, we analyzed the expression of LIF and CCL2 in multiple GBM databases, including TCGA_GBM, CGGA, Rembrandt, and Gravendeel databases. In the four GBM databases, LIF mRNA level and CCL2 mRNA level were positively correlated **(Figure S6A)** and the expression of LIF and CCL2 in mesenchymal subtype was significantly higher than the other subtypes **(Figure 6A and S6B)**. we also investigated the combined expression of LIF and CCL2 in the context of GBM molecular subtype. In TCGA_GBM databases, among 173 GBM cases with LIF^High^/CCL2^High^, 60.1% were mesenchymal subtype. Among 142 GBM cases with LIF^Low^/CCL2^Low^, only 9.2% were mesenchymal subtype, but 64.1% were proneural subtype **(Figure 6B)**, confirming the tight correlation of high levels of LIF and CCL2 with mesenchymal subtype in GBM. In addition, we noticed that 0.5% (1 in 185) GBM cases with LIF^High^/CCL2^High^ were Non G-CIMP, but 22.2% (39 in 176) GBM cases with LIF^Low^/CCL2^Low^ were Non G-CIMP, indicating that LIF and CCL2 was also positively correlated to the development of Non G-CIMP GBM **(Figure S6C)**. We then performed IHC on a tissue microarray containing 169 glioma cases to evaluate the prognostic significance of LIF and CCL2 **(Figure 6C)**. Survival analysis using our own glioma tissue microarray and four GBM databases consistently showed that LIF and CCL2 predicted poor survival of GBM patients **(Figure 6D and S6D)**. Moreover, patients with LIF^High^/CCL2^High^ had significantly shorter overall survival time than those with LIF^Low^/CCL2^Low^ **(Figure 6D)**.

Radiation and chemotherapy with alkylating agents, such as Temozolomide (TMZ), are the most prevalent first-line treatment regimens for GBM treatment. In clinic practice, radiation and chemotherapy can be used individually (single regimen) or conjointly (radio-chemo) 1, 2. We analyzed therapeutic effects of single regimen therapy or radio-chemo therapy using cases of TCGA_GBM database. For single regimen, the survival curve of patients with LIF^High^/CCL2^High^ was similar to that of patients with LIF^High^/CCL2^High^** (Figure S6E)**. For radio-chemo therapy, however, patients with LIF^Low^/CCL2^Low^ had significantly longer survival time than those with LIF^High^/CCL2^High^ **(Figure S6E)**. In addition, both mesenchymal and proneural subtype GBM showed similar response to single regimen therapy, but proneural subtype GBM could benefit from radio-chemo therapy much more than mesenchymal subtype GBM. Interestingly, we noticed that the survival curves of LIF^High^/CCL2^High^ and LIF^Low^/CCL2^Low^ resembled the survival curves of Mesenchymal and Proneural, respectively **(Figure S6E)**. These results implied that LIF and CCL2 might be related with sensitivity of GBM cells to alkylating agents. To further examine this point, we stimulated two GBM cell lines, LN18 and LN229, with recombinant human LIF and CCL2 followed by TMZ treatment. MTT assay showed that LIF and CCL2 stimulation effectively elevated IC_50_ of TMZ in both cell lines compared with vehicle treatment **(Figure S6F)**. We also examined the protein levels of CCL2 and LIF in serum from 2 mGBM patients (mGBM1 and mGBM2) and additional 5 uGBM patients **(Table S13)** through ELISA. The data showed that the serum levels of the two cytokines in mGBM were significantly higher than those in uGBM **(Figure 6E)**. Together, LIF and CCL2 were associated with mesenchymal and Non G-CIMP subtypes and predicted poor prognosis of GBM patients.

### LIF and CCL2 induced mesenchymal-like transcriptome in GBM cells

**Figure 7:**
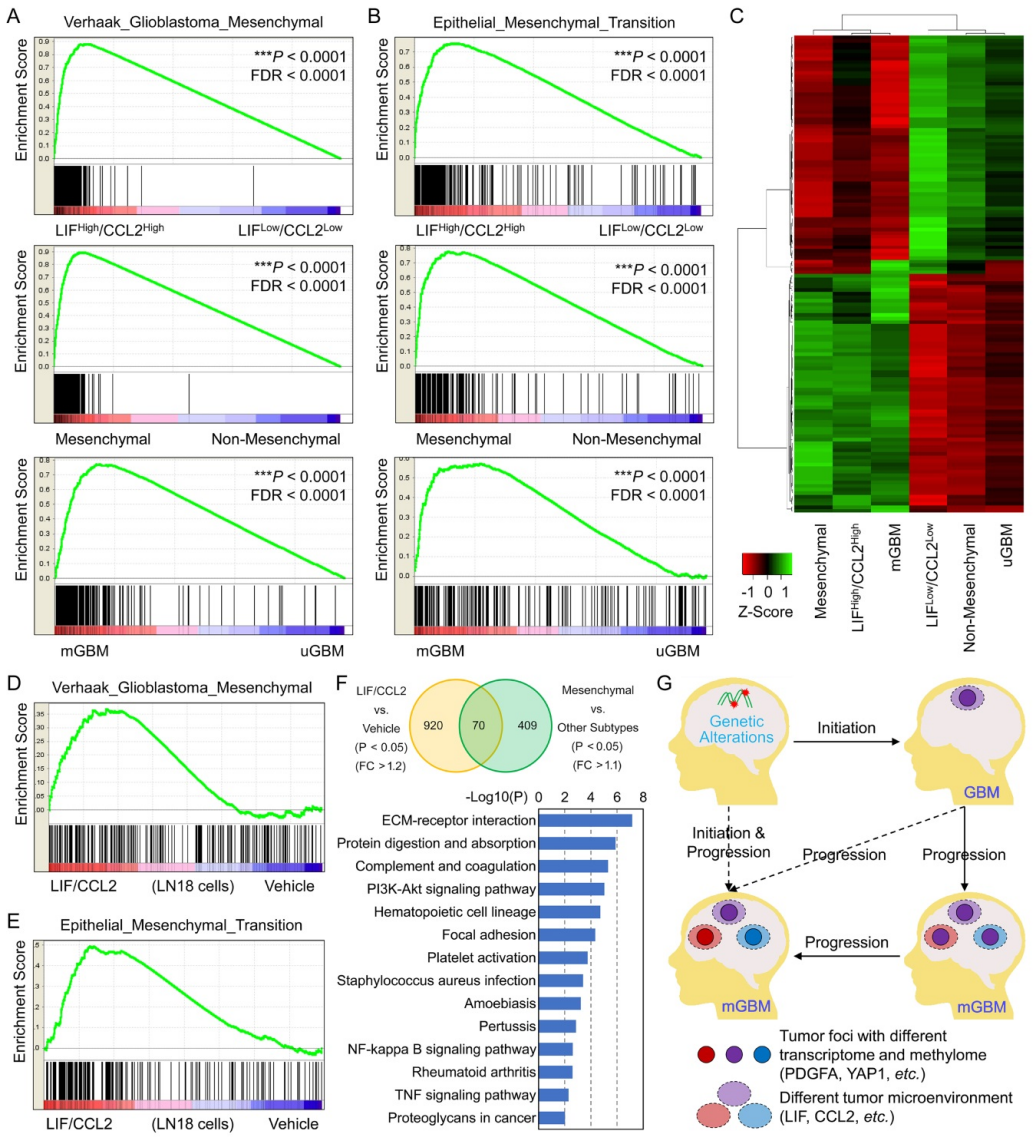
** The correlations among LIF/CCL2 co-expression, mGBM, and mesenchymal GBM.** A) Enrichment of Verhaak_Glioblastoma_Mesenchymal geneset by different GBM phenotypes from TCGA_GBM database through GSEA. B) Enrichment of Hallmark_Epithelial_Mesenchymal_Transition geneset by different GBM phenotypes from TCGA_GBM database through GSEA. C) Cluster heatmap of transcriptomes of different GBM phenotypes from TCGA_GBM database through GSEA. D) Enrichment of Verhaak_Glioblastoma_Mesenchymal geneset by LN18 cells treated with LIF/CCL2 vs. vehicle. E) Enrichment of Hallmark_Epithelial_Mesenchymal_Transition geneset by LN18 cells treated with LIF/CCL2 vs. vehicle. F) Venn diagram of genes significantly upregulated in LN18 cells treated with LIF/CCL2 *vs.* vehicle and mesenchymal subtype *vs.* other subtypes from TCGA_GBM database. FC means Fold Change. G) Schematic diagram of tumor initiation and progression of mGBM.

To evaluate the involvement of LIF and CCL2 in GBM, we performed Gene Set Enrichment Analysis (GSEA) 42, 43 using three TCGA_GBM sub-datasets: mGBM *vs.* uGBM 25, LIF^High^/CCL2^High^ *vs.* LIF^Low^/CCL2^Low^, and mesenchymal subtype *vs.* other subtypes. In all three sub-datasets, GSEA consistently showed that the top one enriched geneset was Verhaak Glioblastoma Mesenchymal in the context of curated genesets (Molecular Signatures Database C2) **(Figure 7A)**, and majority enriched genesets were overlapped among the three sub-datasets **(Figure S7A and Table S14)**. In the context of Hallmark genesets, the enriched genesets by mGBM *vs.* uGBM, LIF^High^/CCL2^High^ *vs.* LIF^Low^/CCL2^Low^, and mesenchymal subtype *vs.* other subtypes were also significantly overlapped **(Figure S7B and Table S15)**, and Epithelial Mesenchymal Transition **(Figure 7B)**, TNFA Signaling Via NFKB, Inflammatory Response, and IL6 JAK STAT3 Signaling were consistently among top-ranked genesets** (Table S15)**. GSEA in the contexts of GO and Oncogenic Signature indicated that the most of enriched genesets were shared by the three sub-datasets **(Figure S7C and S7D and Table S16 and S17)**. We also analyzed TCGA_GBM database and compared the transcriptomic profiles of the three sub-genesets. The data showed that transcriptomic profile of GBM with LIF^High^/CCL2^High^ was significantly correlated with that of mGBM or mesenchymal GBM, but transcriptomic profile of GBM with LIF^Low^/CCL2^Low^ was significantly correlated with that of uGBM or non-mesenchymal GBM** (Figure 7C)**. These data collectively implied that LIF and CCL2 produced similar mRNA expression profiles resembled to that of mGBM or mesenchymal GBM. Moreover, LIF and CCL2 not only prevalent in mGBM but responsible for mesenchymal subtype development in GBM patients.

Finally, we examined the effects of LIF and CCL2 on GBM cells through treating LN18 cells with the mixture of recombinant human LIF and CCL2 followed by RNA-seq for transcriptomic profiling. Analysis on transcriptome data **(Dataset 5)** showed that the treatment indeed resulted in the enrichment of mesenchymal signature **(Figure 7D)**. Additionally, neural and proneural signature genes were enriched by vehicle treatment, but no enrichment of classical **(Figure S7E-G)**. In addition, through GSEA on Hallmark genesets **(Table S18)**, we found the most significantly enriched genesets were Epithelial Mesenchymal Transition **(Figure 7E)**, Inflammatory Response, and IL6 JAK STAT3 Signaling, which was consistent with the results of database analysis above. To describe mesenchymal-related genes induced by LIF and CCL2 treatment in GBM cells, we compared genes upregulated in LIF/CCL2 *vs.* Vehicle (*P*<0.05 and Fold-Change>1.2) and those upregulated in mesenchymal subtype *vs.* other subtypes (*P*<0.05 and Fold-Change>1.1), which revealed 70 genes consistently increased **(Figure 7F and Table S19)**. KEGG analysis indicated these genes were mainly categorized into genesets related with ECM and NFKB **(Figure 7F)**. Thus, our findings supported that LIF and CCL2 were tightly implicated in mesenchymal subtype of GBM.

## Discussion

In this study, we performed WGS, WGBS, and RNA-seq to profile genetic, methylomic and transcriptomic features of mGBM patients. Pathological and genetic examination confirmed that all tumor foci from the three mGBM patients were primary GBM featured with gain of chr.7 and loss of chr.10, lacking IDH1/2 mutations, high frequency of TERT promoter mutation. High frequency of genetic alterations of tumor suppressors (e.g., PTEN and CDKN2A/CDKN2B) and oncogenes (e.g., PDGFA and EGFR) suggested that these cancer driver genes might be responsible for initiation of mGBM but not contribute to the progression of mGBM. Instead, the most significant difference among tumor foci from same patient was microenvironment-related genes (such as LIF and CCL2), emphasizing the critical involvement of these genes in various tumor evolution routes **(Figure 7G)**. For mGBM1 and mGBM3, all tumor foci shared critical oncogenic alterations of GBM, including gain of chromosome 7, loss of chromosome 10, and many significantly mutated genes, supporting their monoclonal origin for mGBM1 and 3, which is consistent with report by Abou-El-Ardat *et al.* 6. For mGBM2, focus A and B showed consistent chromosomal alterations, but focus C lacked of chromosomal alterations, which might imply different origin of mGBM2_C with A and B, or differentially evolved at very early stage of GBM initiation. Similar case has also been reported by Lombardi *et al.* 44.

Whole genome methylation, especially methylation alterations in promoter area, pivotally regulates gene expression. Through analysis on the methylomes of the nine tumor foci, we found that chr.22, 17, and 16 were consistently hypomethylated in all tumor foci from the three mGBM patients. GO analysis showed that promoter methylation levels of olfactory transduction-related genes were significantly altered in the three mGBM patients. It has been reported that olfactory-related genes are actually involved in GBM development and could serve as prognostic markers for GMB 45-47. Methylomic analysis also showed critical involvement of Hippo/YAP1 signaling axis. Analysis on public glioma databases revealed higher expression of YAP1 in mesenchymal subtype than other subtypes and shortened survival of patients with high YAP1 expression compared to those with low YAP1 expression. Our previous work also showed critical involvement of YAP1 in GBM 48, 49. Thus, our findings in mGBM implied that the promoter methylation might be responsible for the regulation of olfactory-related genes and Hippo/YAP1 pathway in GBM.

Analysis on transcriptomic profiles of the nine tumor foci suggested that gene clusters of different tumor foci from same patients were distinguished from each other. As a result, tumor foci from same patients showed varied molecular subtypes and predicted varied prognosis, further confirming intratumor heterogeneity. Interestingly, we noticed that ECM and immune system were pivotally involved in mGBM evolution. Genes related with ECM and immune response were found to be dramatically changed among tumor foci from same patients. It has been known that ECM is altered in GBM and plays critical role in invasive growth of GBM cells 50-52. Moreover, immune microenvironment is responsible for development of mesenchymal subtype in GBM 8, 29 and results in treatment resistance 32, 33. We also noticed that interaction with neuron and normal synapse functions were involved in evolution of tumor foci. It has been found that there is direct communication between neurons and glioma cells through synapses and the integration of synaptic and electrical into neural circuits promotes glioma progression 53, 54.

Our work intriguingly revealed that two cytokines, LIF and CCL2, were positively correlated in expression levels and produced a phenotype resembling mesenchymal subtype GBM with poor prognosis. It is hard to define molecular subtype of GBM cell line, but our work indicated that treatment of CCL2 and LIF altered gene expression profiles in GBM cells, which significantly enriched signature genes of mesenchymal subtype, but failed to enrich signature genes of other molecular subtypes. LIF and CCL2 have been found to be involved in glioma 39, 55, 56, but their relationships with molecular subtypes of GBM have not yet been described. Herein, we noticed that high expression of both cytokines was tightly correlated with mesenchymal subtype but low expression of both cytokines was significantly related with proneural subtype. Previous reports indicated that mGBM mainly assumes mesenchymal subtype almost half (47%) the mGBM belonged to the mesenchymal subtype, and examined 6 tumor foci and showed that 1 was associated with mesenchymal and the other 5 were associated with both mesenchymal and classical subtypes 6, 25. Thus, LIF and CCL2 might be responsible for high percentage of mesenchymal subtype tumor focus in mGBM and the two cytokines could help diagnosis of molecular subtypes of GBM in histopathological examination.

Intertumor and intratumor heterogeneity of GBM is believed to render resistance of GBM patients to routine treatments 18, 57. According to our results, the mGBM reported here was highly heterogenous and might be resistant to radio and chemotherapy. Consistently, the patient reported here developed two new neoplasms in brain at 35-day after surgery and progressed to form two more neoplasms by 165-day after surgery. Thus, mGBM patients assumed high recurrence rate possibly due to the high heterogeneity.

We should mention that the size of patient samples in this study were limited and the functions of CCL2 and LIF in mGBM have not been examined with animal models, which were worth further pursuing in the following study to delineate the mechanisms on the formation of mGBM. Altogether, the integrative omics analyses confirmed existence of various GBM molecular subtypes from same patients and revealed pivotal impact of tumor microenvironment on molecular subtypes during tumor progression. Key genetic drivers were actually shared by all tumor foci from same patients, and thus might not be responsible for tumor evolution. Instead, extracellular components, including LIF, CCL2, PDGFA, Hippo/YAP1, as well as, immune responses and angiogenesis, might pivotally participate in development of intratumor heterogeneity through remodeling methylome and transcriptome of tumor foci from same mGBM patients.

## Methods

### Patient and samples

The three patients were hospitalized in the Department of Neurosurgery, Xinqiao Hospital of Third Military Medical University in 2019 and 2020 and performed operation after a clear evaluation 3 days later. This study was approved by the Medical Ethical Committees of Xinqiao Hospital and Southwest Hospital, Third Military Medical University. Written informed consents were obtained from the patients. Glioma tissue microarray (HBraG169Su01) was purchased from Shanghai Outdo Biotech CO., LTD. (http://www.superchip.com.cn/index.html).

*More details on Methods were described in supplementary materials.*

## Supplementary Material

Supplementary figures and materials and methods.

Supplementary table 1.

Supplementary dataset 1.

Supplementary dataset 2.

Supplementary dataset 3.

Supplementary dataset 4.

Supplementary dataset 5.

## Abbreviations

Chr.: chromosome
ECM: extracellular matrix
GO: gene ontology
GSEA: gene Set Enrichment Assay
GBM: glioblastoma
mGBM: multifocal GBM
PCA: principal component analysis
WGBS: whole genome bisulfite sequencing
WGS: whole-genome sequencing
