# Supplementary material for: Comprehensive omics analyses profile genesets related with tumor heterogeneity of multifocal glioblastomas and reveal LIF/CCL2 as biomarkers for mesenchymal subtype
Source: Theranostics. 2022 Jan 1;12(1):459–73. doi: 10.7150/thno.65739 (PMC8690928; doi:10.7150/thno.65739)
Supplement: Supplementary file 1 — Supplementary figures and materials and methods. [file thnov12p0459s1.pdf]

Supplementary Figures:

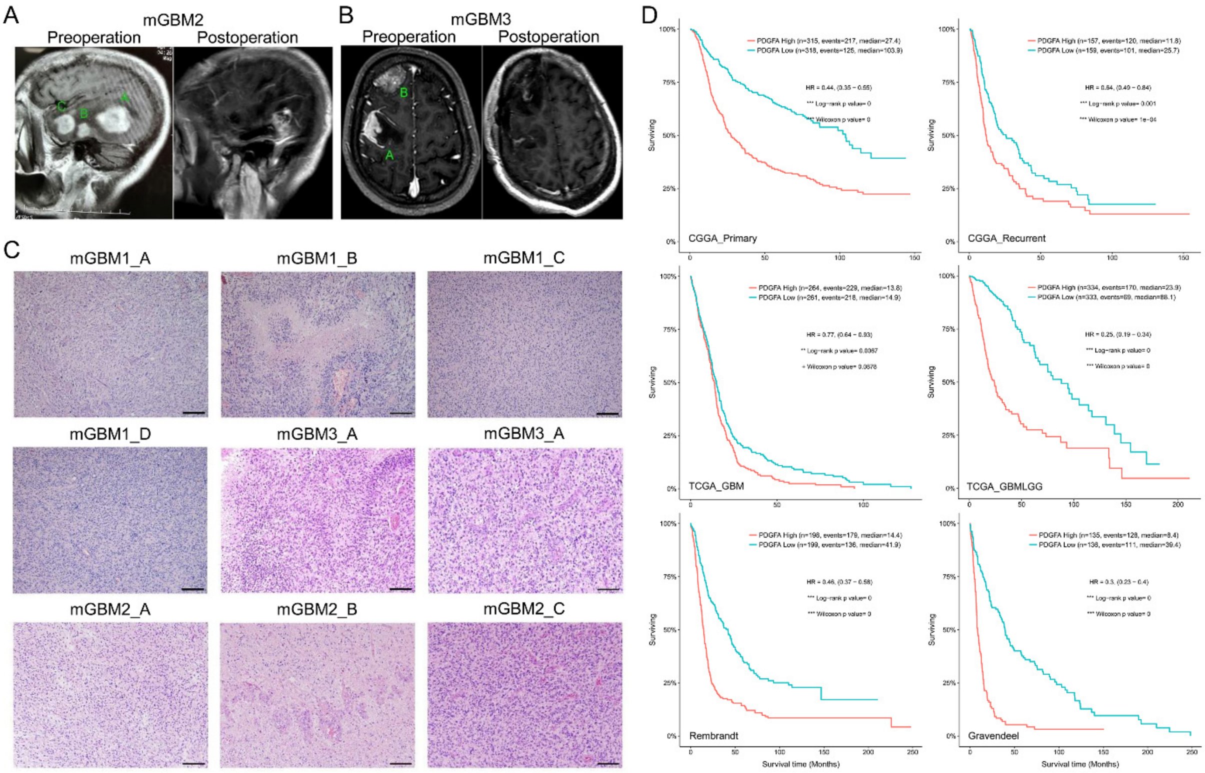

Figure S1. Histopathological examination of three mGBM patients.

A) Representative MRI images on the mGBM2 patient. A, B, and C represent three tumor foci.

B) Representative MRI images on the mGBM3 patient. A and B represent two tumor foci.

C) Representative HE staining of tumor foci from three mGBM patients. Scale bars: 100 μm.

D) Kaplan-Meier survival analysis of PDGFA mRNA from multiple glioma databases.

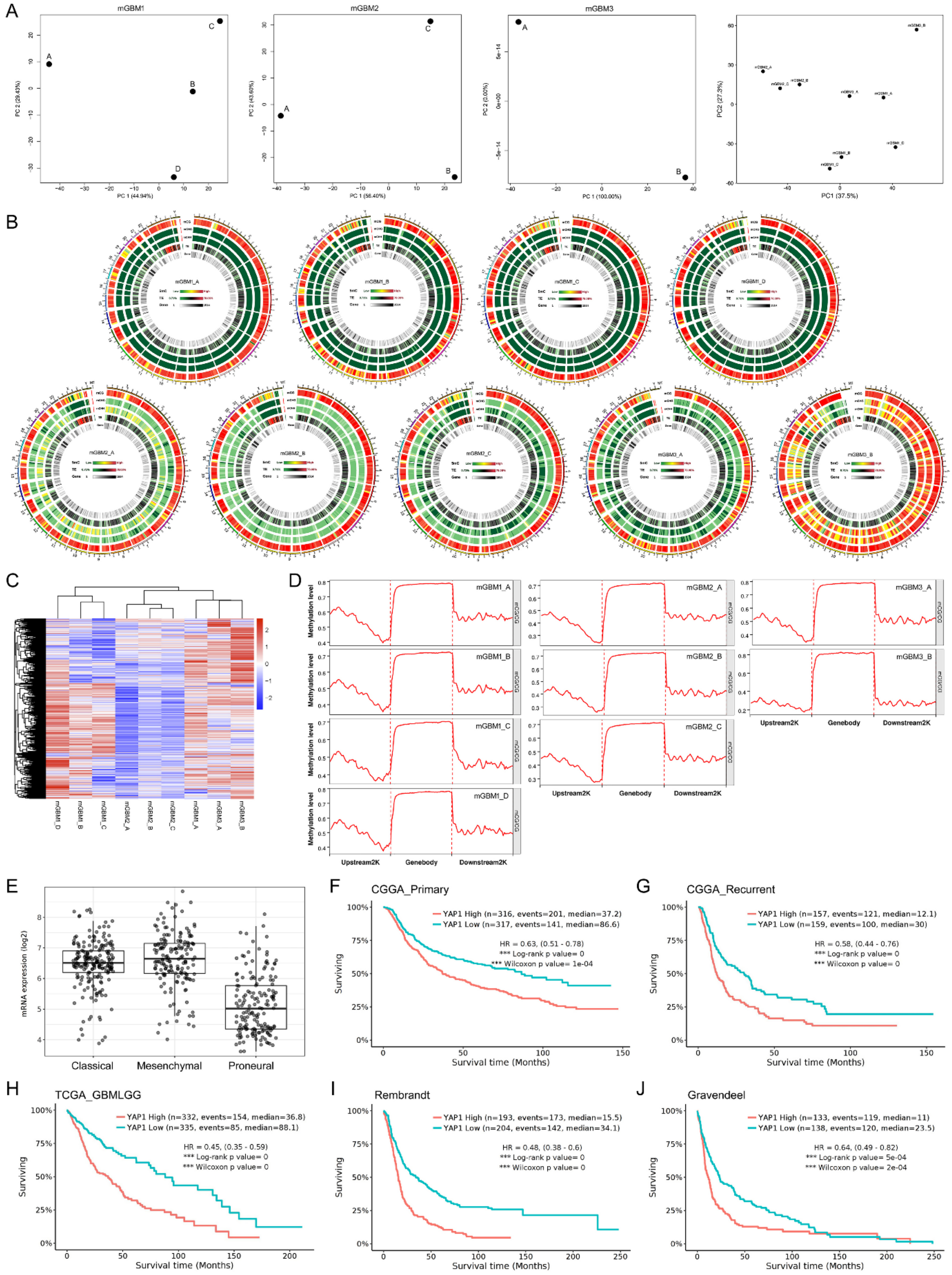

Figure S2. Whole Genome Methylomic analysis on tumor foci from three mGBM patients.

A) Principal component analysis of methylomes of tumor foci from the three mGBM patients.

B) Circos plot of distribution of cytosine with 5-methylation with different motif. mCG: methylated CG motif; mCHG: methylated C(A/T/C)G motif; mCHH: methylated C(A/T/C)(A/T/C) motif.

C) Clusters of nine tumor foci from three mGBM patients.

D) Analysis on CpG methylation levels in various DNA regions of tumor foci from the three mGBM samples.

E) mRNA Expression of YAP1 in TCGA_GBM database according to molecular subtypes

F) -J) Kaplan-Meier survival analysis of YAP1 mRNA from multiple glioma databases.

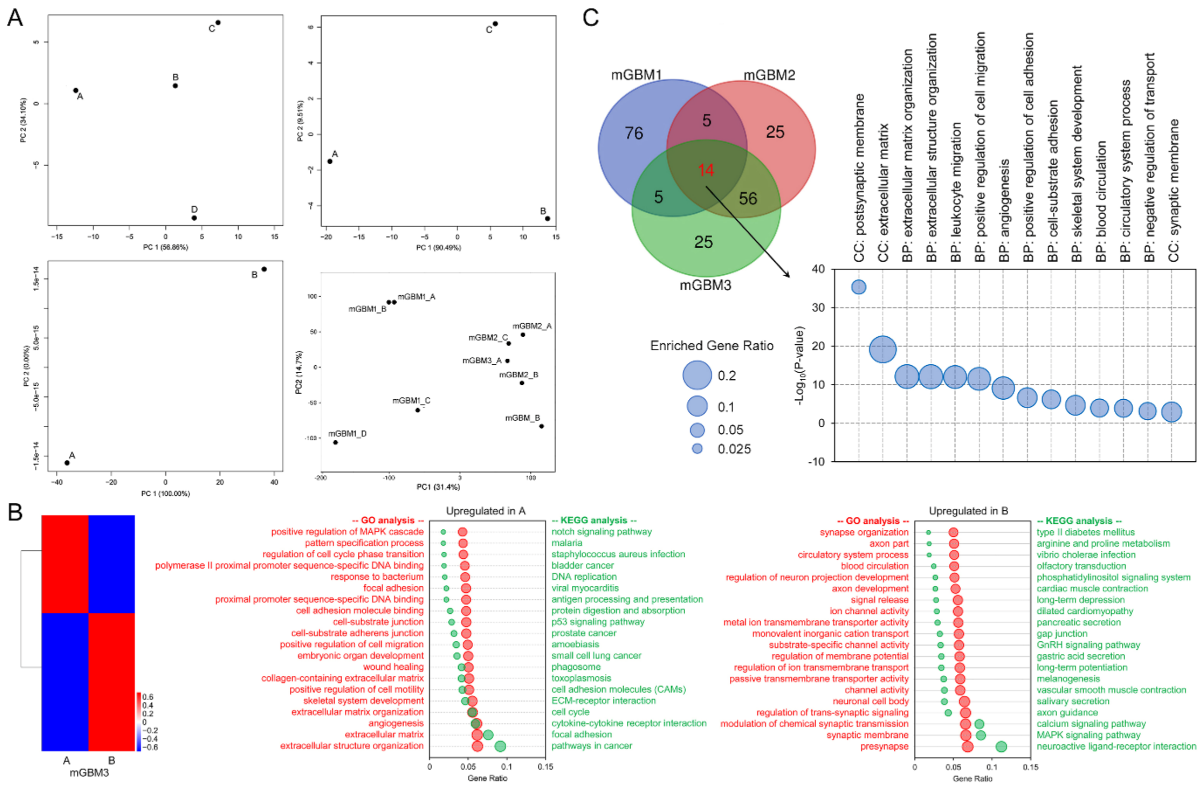

Figure S3. Transcriptomic analysis on tumor foci from three mGBM patients

A) Principal component analysis of transcriptomes of tumor foci from the three mGBM patients.

B) Analysis on transcriptomes of tumor foci from mGBM3. Left: Heatmap diagram showing the cluster of gene expression in different tumor foci from mGBM3. Middle: Gene enrichment of significantly upregulated mRNA in mGBM3_A compared with mGBM3_B. Right: Gene enrichment of significantly upregulated mRNA in mGBM3_B compared with mGBM3_A.

C) Venn diagram and scatter graph showing 14 GO genesets consistently enriched by significantly altered genes in all three mGBM samples.

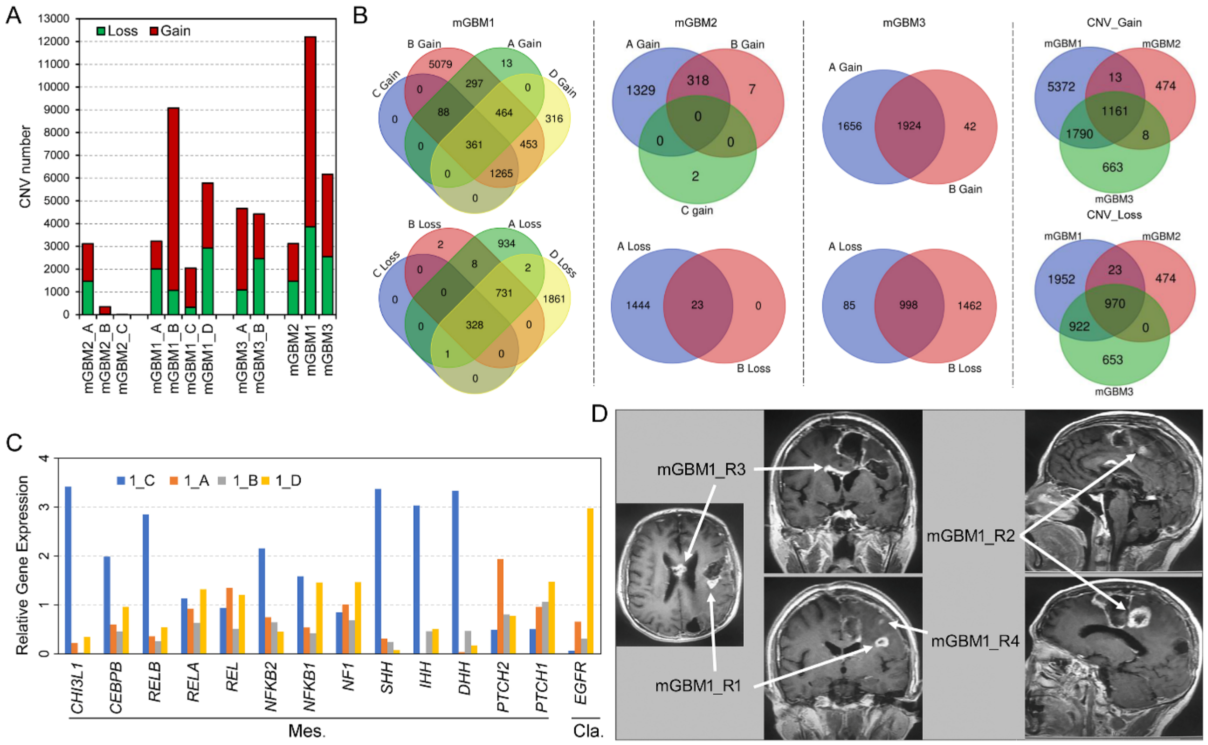

Figure S4. Expression of signature genes of molecular subtype and prognosis of mGBM1.

A) CNV (gain and loss) counts in different tumor foci from same mGBM sample as well as in the three mGBM samples.

B) Venn diagram showing shared and tumor focus-specific CNV (gain and loss) counts in different tumor foci from same mGBM sample as well as in the three mGBM samples.

C) mRNA levels of core genes for mesenchymal and classical subtypes from four tumor foci of mGBM1.

D) The MRI images showing newly developed tumor foci (mGBM1_R1, R2, R3, and R4) after surgery and standard radio- and chemo-therapy.

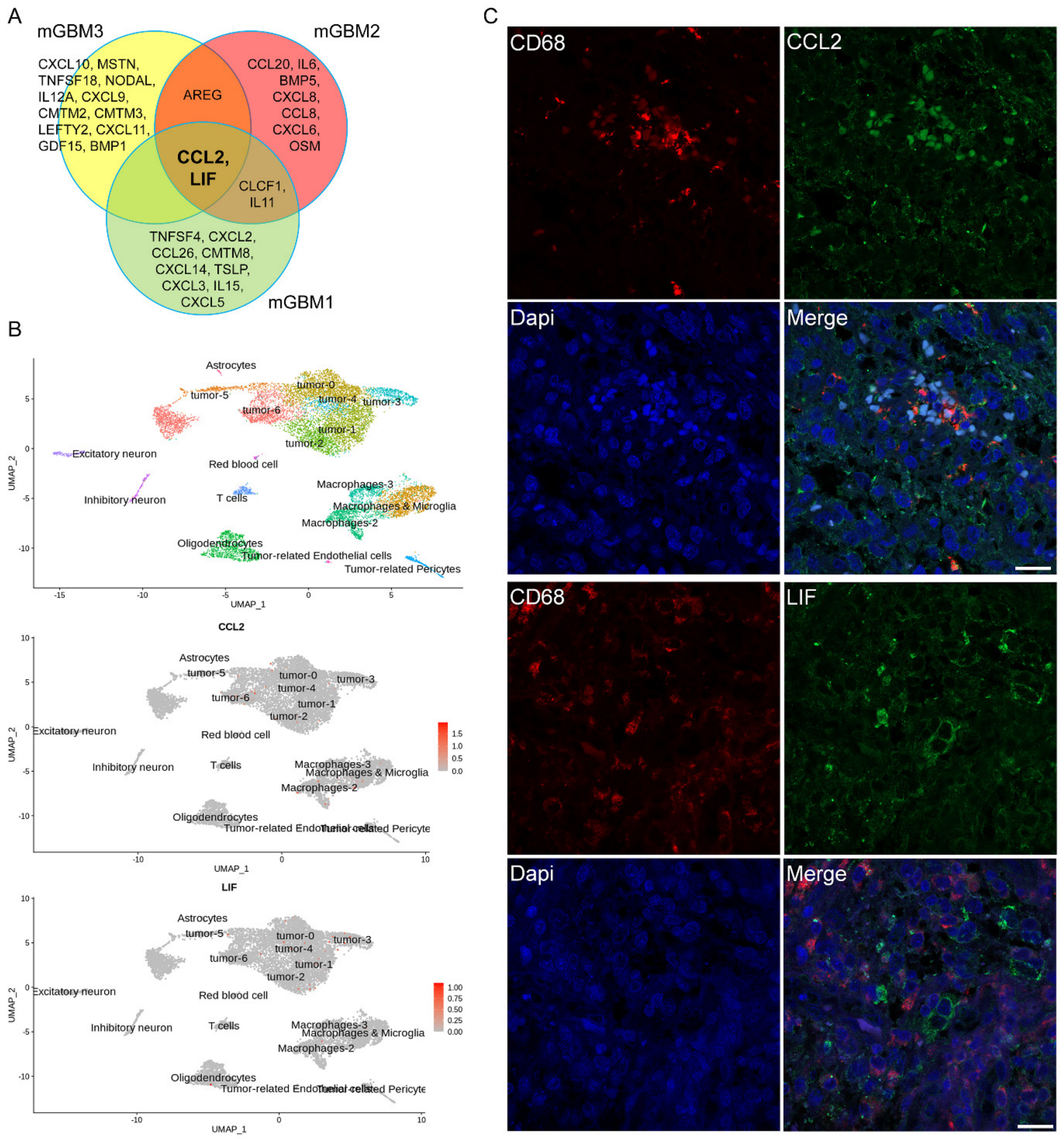

Figure S5. Expression of signature genes of molecular subtype and prognosis of mGBM1.

A) Venn diagram of cytokines upregulated in tumor focus of mesenchymal subtype compared to those of non-mesenchymal subtype in same mGBM samples, indicating CCL2 and LIF as consistently upregulated genes in the three mGBM samples

B) UMAP analysis on single cell sequencing of GBM showing cell types (top), CCL2 mRNA level in different types of cells (middle), and LIF mRNA level in different types of cells (bottom).

C) Representative Immunofluorescence images of indicated proteins on mGBM1_C. Dapi is used to show cell nuclei. Scale bars: 20 μm.

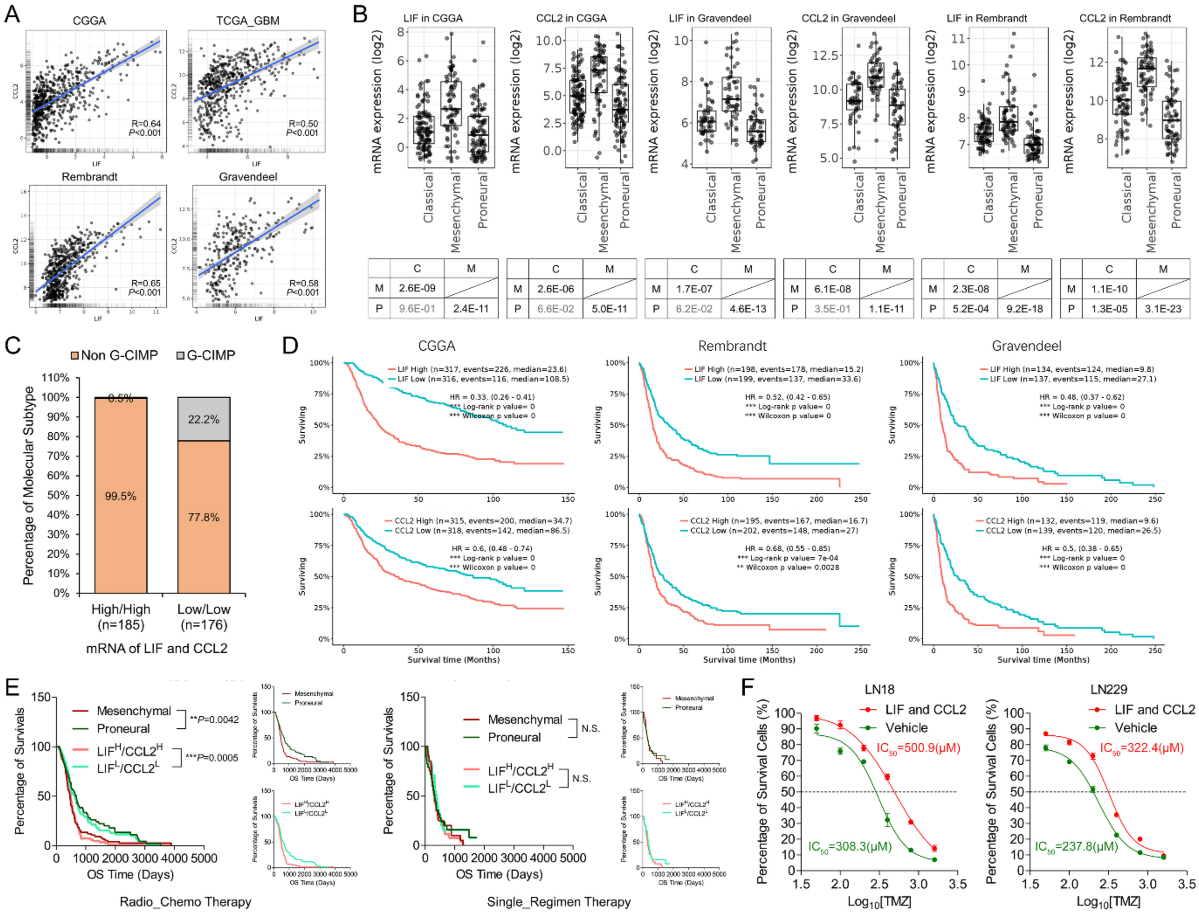

Figure S6. Expression of signature genes of molecular subtype and prognosis of mGBM1.

A) Pearson correlation between LIF mRNA and CCL2 mRNA in different GBM public databases.

B) mRNA expression of LIF and CCL2 in different molecular subtypes using three GBM databases.

C) Percentage of G-CIMP and Non G-CIMP status of GBM with $LIF^{High}/CCL2^{High}$ and $LIF^{Low}/CCL2^{Low}$, respectively.

D) Kaplan-Meier survival analysis on mRNA levels of LIF or/and CCL2 in three GBM databases.

E) Kaplan-Meier survival analysis of radio-chemo (Left) or single regimen (Right) treatments on patients with (LIF mRNA)^{High}/(CCL2 mRNA)^{High} vs. (LIF mRNA)^{Low}/(CCL2 mRNA)^{Low} or Mesenchymal subtype vs. Proneural subtype using TCGA_GBM database.

F) Growth curve of LN18 and LN229 cells in response to TMZ for 72 hours with co-treatment of LIF/CCL2 or Vehicle.

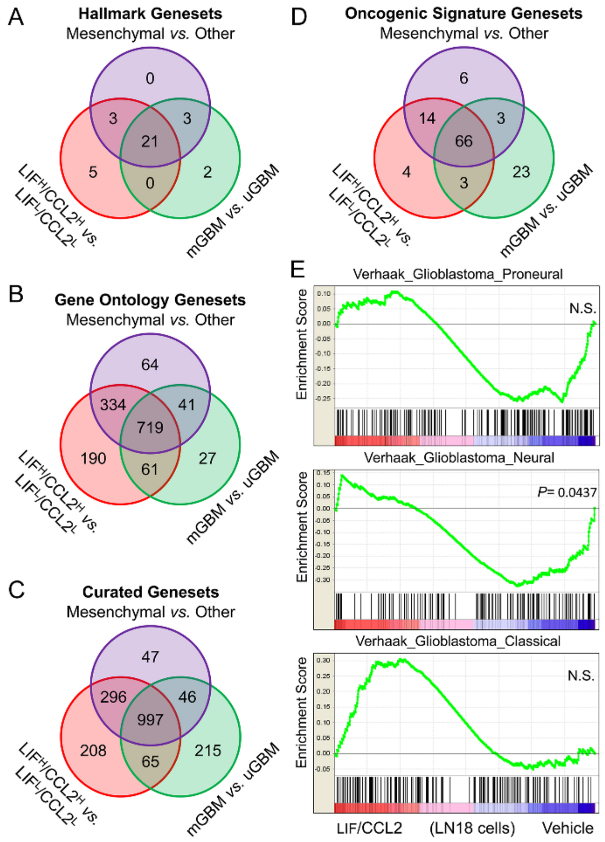

Figure S7. GSEA of different GBM phenotypes from TCGA_GBM database.

A) - D) Venn diagram of enriched genesets by different GBM phenotypes in the context of indicated gensets.

E) Enrichment of indicated geneset according to Verhaak GBM molecular subtypes by LN18 cells treated with LIF/CCL2 vs. vehicle.

Supplementary Materials and Methods

RNA sequencing and data processing

(1) Library preparation for Transcriptome sequencing

Total RNA of cells was extracted using TRIzol reagent (15596026 ThermoFisher, USA) according to manufacturer's instruction. A total amount of 2 µg RNA per sample was used as input material for the RNA sample preparations. Sequencing libraries were generated using NEBNext® UltraTM RNA Library Prep Kit for Illumina® (NEB, USA) following manufacturer's recommendations and index codes were added to attribute sequences to each sample. Briefly, mRNA was purified from total RNA using poly-T oligo-attached magnetic beads. Fragmentation was carried out using divalent cations under elevated temperature in NEBNext First Strand Synthesis Reaction Buffer(5X). First strand cDNA was synthesized using random hexamer primer and M-MuLV Reverse Transcriptase (RNase H-). Second strand cDNA synthesis was subsequently performed using DNA Polymerase I and RNase H. Remaining overhangs were converted into blunt ends via exonuclease/polymerase activities. After adenylation of 3' ends of DNA fragments, NEBNext Adaptor with hairpin loop structure were ligated to prepare for hybridization. In order to select cDNA fragments of preferentially 150~200 bp in length, the library fragments were purified with AMPure XP system (Beckman Coulter, Beverly, USA). Then adaptor-ligated cDNA at 37°C for 15 min followed by 5 min at 95 °C before PCR. Then PCR was performed with Phusion High-Fidelity DNA polymerase, Universal PCR primers and Index (X) Primer. At last, PCR products were purified (AMPure XP system) and library quality was assessed on the Agilent Bioanalyzer 2100 system.

(2) Clustering and sequencing

The clustering of the index-coded samples was performed on a cBot Cluster Generation System using TruSeq PE Cluster

Kit v3-cBot-HS (Illumia) according to the manufacturer's instructions. After cluster generation, the library preparations were sequenced on an Illumina Hiseq platform and 125 bp/150 bp paired-end reads were generated.

(3) Quality control

Raw data (raw reads) of fastq format were firstly processed through in-house perl scripts. In this step, clean data (clean reads) were obtained by removing reads containing adapter, reads containing ploy-N and low-quality reads from raw data. At the same time, Q20, Q30 and GC content the clean data were calculated. All the downstream analyses were based on the clean data with high quality.

(4) Reads mapping to the reference genome

Reference genome and gene model annotation files were downloaded from genome website directly. Index of the reference genome was built using Bowtie v2.2.3 and paired-end clean reads were aligned to the reference genome using Hisat2 v2.0.5. We selected Hisat2 as the mapping tool for that Hisat2 can generate a database of splice sets based on the gene model annotation file and thus a better mapping result than other non-splice mapping tools.

(5) Quantification of gene expression level

HTSeq was used to count the reads numbers mapped to each gene. And then FPKM of each gene was calculated based on the length of the gene and reads count mapped to this gene. FPKM, expected number of Fragments Per Kilobase of transcript sequence per Millions base pairs sequenced, considers the effect of sequencing depth and gene length for the reads count at the same time, and is currently the most used method for estimating gene expression levels [1].

(6) Differential expression analysis

Differential expression analysis was performed using the edgeR package. edgeR is one of the most popular Bioconductor packages for assessing differential expression in RNA-seq data. It is based on the negative binomial (NB) distribution and it models the variation between biological replicates through the NB dispersion parameter. This method is immediately able to handle complex experimental designs. Genes with an adjusted P-value <0.05 found by DESeq were assigned as differentially expressed [2]. Differential expression analysis of two conditions was performed using the DEGSeq R package. The P values were adjusted using the Benjamini & Hochberg method. Corrected P-value of 0.005 and log2(Fold change) of 1 were set as the threshold for significantly differential expression.

Whole Genome Sequencing

(1) DNA Quantification & Qualification

The quality of isolated genomic DNA was verified by using these two methods in combination: DNA degradation and contamination were monitored on 1% agarose gels. DNA concentration was measured by Qubit® DNA Assay Kit in Qubit® 2.0 Flurometer (Invitrogen, USA).

(2) Library Preparation

A total amount of 0.5 μg DNA per sample was used as input material for the DNA library preparations. Sequencing library was generated using Truseq Nano DNA HT Sample Prep Kit (Illumina USA) following manufacturer's recommendations and index codes were added to each sample. Briefly, genomic DNA sample was fragmented by sonication to a size of 350 bp. Then DNA fragments were endpolished, A-tailed, and ligated with the full-length adapter for Illumina sequencing, followed by further PCR amplification. After PCR products were purified (AMPure XP system), libraries were analyzed for size distribution by Agilent 2100 Bioanalyzer and quantified by real-time PCR (3nM).

(3) Clustering & Sequencing

The clustering of the index-coded samples was performed on a cBot Cluster Generation System using Hiseq X PE Cluster Kit V2.5 (Illumina) according to the manufacturer's instructions. After cluster generation, the DNA libraries were sequenced on Illumina Hiseq platform and 150 bp paired-end reads were generated.

(4) Quality Control

The original fluorescence image files obtained from Hiseq platform are transformed to short reads (Raw data) by base calling and these short reads are recorded in FASTQ format, which contains sequence information and corresponding sequencing quality information. Sequence artifacts, including reads containing adapter contamination, low-quality

nucleotides and unrecognizable nucleotide (N), undoubtedly set the barrier for the subsequent reliable bioinformatics analysis. Hence quality control is an essential step and applied to guarantee the meaningful downstream analysis.
The steps of data processing were as follows:

- Discard paired reads if either one read contains adapter contamination (>10 nucleotides aligned to the adapter, allowing ≤ 10% mismatches);
- Discard paired reads if more than 10% of bases are uncertain in either one read;
- Discard paired reads if the proportion of low quality (Phred quality <5) bases is over 50% in either one read.

All the downstream bioinformatics analyses were based on the high-quality clean data, which were retained after these steps. At the same time, QC statistics including total reads number, raw data, raw depth, sequencing error rate and percentage of reads with Q30 (the percent of bases with phred-scaled quality scores greater than 30) were calculated and summarized.

(4) Reads Mapping to Reference Sequence

Valid sequencing data was mapped to the reference human genome (UCSC hg19) by Burrows-Wheeler Aligner (BWA) software [3] to get the original mapping results stored in BAM format. If one or one paired read(s) were mapped to multiple positions, the strategy adopted by BWA was to choose the most likely placement. If two or more most likely placements presented, BWA picked one randomly. Then, SAMtools [4] and Picard (http://broadinstitute.github.io/picard/) were used to sort BAM files and do duplicate marking, local realignment, and base quality recalibration to generate final BAM file for computation of the sequence coverage and depth. Mapping step was very difficult due to mismatches, including true mutation and sequencing error, and duplicates resulted from PCR amplification. These duplicate reads were uninformative and shouldn't be considered as evidence for variants. We used Picard to mark these duplicates for follow up analysis.

(5) Variant Calling

Samtools [4] mpileup and bcftools were used to do variant calling and identify SNP, InDels. Control-FREEC [5] was utilized to do CNV detection, while Crest [6] was specialized for SV discovery.

(6) Functional Annotation

Functional annotation was very important because the link between genetic variations and diseases would be clarified in this step. ANNOVAR [7] was performed to do annotation for VCF (Variant Call Format) obtained in the previous effort. dbSNP, 1000 Genome and other related existing databases were applied to characterize the detected variants. Given to the significance of exonic variants, gene transcript annotation databases, such as Consensus CDS, RefSeq, Ensembl and UCSC, were also included to determine amino acid alternation. Annotation content contained the variant position, variant type, conservative prediction, etc. These annotation results would help to locate disease causal mutant. The details of annotation were exhibited in supplemented material.

(7) Somatic Mutation Calling

The somatic SNV was detected by muTect [8], the somatic InDel by Strelka [9], and the somatic structural variants (SV) by CREST [6]. Control-FREEC was used to detect somatic CNV [5].

Whole genome bisulfite sequencing

(1) DNA quantification and qualification

Genomic DNA degradation and contamination was monitored on agarose gels. DNA purity was checked using the NanoPhotometer® spectrophotometer (IMPLEN, CA, USA). DNA concentration was measured using Qubit® DNA Assay Kit in Qubit® 2.0 Flurometer (Life Technologies, CA, USA).

(2) Library preparation and quantification

A total amount of 100 nanogram genomic DNA spiked with 0.5 ng lambda DNA were fragmented by sonication to 200-300bp with Covaris S220. These DNA fragments were treated with bisulfite using EZ DNA Methylation-GoldTM Kit (Zymo Research), and the library kit was Accel -NGS Methyl -Seq DNA Library Kit for illumina. Library concentration was quantified by Qubit® 2.0 Flurometer (Life Technologies, CA, USA) and quantitative PCR, and the insert size was assayed

on Agilent Bioanalyzer 2100 system.

(3) Quality control

The library preparations were sequenced on an Illumina Hiseq XTen or Novaseq platform and 125 bp/150 bp paired -end reads were generated. Image analysis and base calling were performed with Illumina CASAVA pipeline, and finally 125bp/150bp paired-end reads were generated. First, we use FastQC (fastqc_v0.11.5) to perform basic statistics on the quality of the raw reads. Then, those reads sequences produced by the Illumina pipleline in FASTQ format were pre-processed through Trimmomatic (Trimmomatic-0.36) software use the parameter (SLIDINGWINDOW: 4:15 ; LEADING:3, TRAILING:3 ; ILLUMINACLIP: adapter.fa: 2: 30: 10 ; MINLEN:36) The remaining reads that passed all the filtering steps was counted as clean reads and all subsequent analyses were based on this. At last, we use FastQC to perform basic statistics on the quality of the clean data reads.

(4) Reference data preparation before analysis

Before the analysis, we prepare the reference data for the species we study, which includes the reference sequence fasta file, the annotation file in gtf format, the GO annotation file, the description file and the gene region file in bed format. As for the bed files, we predict repeats through RepeatMasker, followed by getting CGI track from a genome use cpg IslandExt.

(5) Reads mapping to the reference genome

Bismark software (version 0.16.3) [10] was used to perform alignments of bisulfite-treated reads to a reference genome (-X 700 --dovetail). The reference genome was firstly transformed into bisulfite-converted version (C-to-T and G-to-A converted) and then indexed using bowtie2 [11]. Sequence reads were also transformed into fully bisulfite-converted versions (C-to-T and G-to-A converted) before they are aligned to similarly converted versions of the genome in a directional manner. Sequence reads that produce a unique best alignment from the two alignment processes (original top and bottom strand) are then compared to the normal genomic sequence and the methylation state of all cytosine positions in the read is inferred. The same reads that aligned to the same regions of genome were regarded as duplicated ones. The sequencing depth and coverage were summarized using deduplicated reads. The results of methylation extractor (bismark_methylation_extractor, - no_overlap) were transformed into bigWig format for visualization using IGV browser. The sodium bisulfite non-coversion rate was calculated as the percentage of cytosine sequenced at cytosine reference positions in the lambda genome.

(6) Estimating methylation level

To identify the methylation site, we modeled the sum Mc of methylated counts as a binomial (Bin) random variable with methylation rate r:

mC ~ Bin (mC + umC * r)

In order to calculate the methylation level of the sequence, we divided the sequence into multiple bins, with bin size is 10kb. The sum of methylated and unmethylated read counts in each window were calculated. Methylation level (ML) for each window or C site shows the fraction of methylated Cs, and is defined as:

ML(C) = reads(mC) / [reads(mC) + reads(C)]

Calculated ML was further corrected with the bisulfite non-conversion rate according to previous studies [12]. Given the bisulfite nonconversion rate r, the corrected ML was estimated as:

$$ML_{(corrected)} = (ML - r) / (l - r)$$

(7) Differentially methylated analysis

Differentially methylated regions (DMRs) were identified using the DSS software [13-15]. The core of DSS is a new dispersion shrinkage method for estimating the dispersion parameter from Gamma-Poisson or Beta-Binomial distributions. DSS possess three characteristics to detect DMRs. First, spatial correlation. Proper utilization of the information from neighboring Cytosine sites can help improve estimation of methylation levels at each Cytosine site, and hence improve DMR detection. Second, the read depth of the Cytosine sites provides information on precision that can be exploited to improve statistical tests for DMR detection. Finally, the variance among biological replicates provides information necessary for a valid statistical test to detect DMRs，when there is no biological replicate, DSS combining data from nearby Cytosine sites and using them as ‘pseudo-replicates’ to estimate biological variance at specific locations.

According to the distribution of DMRs through the genome, we defined the genes related to DMRs as genes whose gene body region (from TSS to TES) or promoter region (upstream 2kb from the TSS) have an overlap with the DMRs.

Cell culture

Human GBM cell line LN18 and LN229 was obtained from the ATCC (www.ATCC.org) and maintained at 37°C in a 5% CO2 incubator DMEM/F12 plus 10% fetal bovine serum. The cell line was characterized as mycoplasma negative and validated by STR DNA fingerprinting using the AmpFLSTR Identifiler kit (#4322288, ThermoFisher) according to the manufacturer's instructions semiannually. The STR profiles were compared with known ATCC fingerprints and matched known DNA fingerprints. Addition of LIF and CCL2 was performed according to product datasheet.

TMZ IC_{50} calculation

5,000 cells were seeded in 96-well plate for overnight growth and then TMZ with series concentrations was added into the wells with the presence of LIF/CCL2 or PBS as vehicle. After 72 h growth, the cell viability was measured through CCK-8 assay and the IC_{50} was calculated using GraphPad Prism 5 software.

ELISA for serum LIF and CCL2 from GBM patients

The kits were purchased from R&D systems (Quantikine® ELISA Human LIF Immunoassay #DLF00B and Quantikine® ELISA Human CCL2/MCP-1 Immunoassay #DCP00), and the serum from seven patients (mGBM1, mGBM2, and five uGBM patients) **(Table S1)** were pre-treated followed by ELISA according to the manufacturer's instructions. Standard curve was established with recombinant human LIF and recombinant human CCL2.

Immunohistochemistry

The immunohistochemistry (IHC) was performed according to our previous study [16]. The evaluation of IHC staining was performed as below. Five most characteristic high-power fields (× 400 magnification) per tissue section were manually selected using an Olympus BX51 microscope (Olympus, Tokyo, Japan). The staining results were calculated using ImageJ software based on H-Score method, i.e., signal intensity × positive cell percentage. Anti-YAP (#14074) was from Cell Signaling Technology. Anti-CCL2 (#sc-32771) was from Santa Cruz Biotechnology. Anti-LIF (#ab138002), anti-CD31 antibody (ab9498), anti-CD163 (ab182422), anti-CD4 (ab133616), and anti-CD8 (ab4055) were from abcam. Recombinant human LIF (#300-05) and recombinant human CCL2 (#300-04) were from PeproTech.

Immunofluorescence

Formalin-fixed GBM tissues were embedded in paraffin wax and sectioned at a thickness of 3 μm. Sections were deparaffinized and rehydrated using a graded series of ethanol solutions. After H_2O_2 blocking and antigen retrieval, the sections were incubated with Immunostaining blocking serum (Beyotime, P0102) for 1 hr and then incubated with diluted primary antibodies at 4°C overnight. The sections were further incubated with fluorophore-conjugated secondary antibody for 60 min. The cell nuclei were counterstained with Antifade Reagent with DAPI (Cell Signaling Technology #8961) for 15min. Images were obtained using a Zeiss LSM900 laser confocal microscope. Primary antibodies listed as follows: anti-GFAP(Cell Signaling Technology, #12389, 1:200), anti-OLIG2 (R&D Systems, #AF2418, 1:200); anti-CCL2 (Santa Cruz, sc-32771, 1:100), and anti-LIF (Abcam, ab138002, 1:150). Fluorophore-conjugated secondary antibody listed as follows: Donkey anti-Goat IgG H&L, Alexa Fluor Plus 555 (Invitrogen, A32816, 1:500); Donkey anti-Rabbit IgG H&L, Alexa Fluor Plus 555 (Invitrogen, A32794, 1:500); Donkey anti-Mouse IgG H&L, Alexa Fluor Plus 488 (Invitrogen, A32766, 1:500) and Donkey Anti-Rat IgG H&L, Alexa Fluor Plus 488 (Abcam, ab150153, 1:500).

Statistical analysis

GBM and glioma databases were downloaded from **https://xenabrowser.net/datapages/** and online analysis was done

on **gliovis.bioinfo.cnio.es/** or **http://gepia2.cancer-pku.cn/#index**. DAVID Bioinformatics Resources [17] and Gene Set Enrichment Assay (GSEA) (P < 0.05, FDR < 0.25) [18] were used to analyze our data. The statistical significance was determined by Student's t test and P < 0.05 was considered statistically significant. Kaplan-Meier survival plot sand log-rank statistics were used to evaluate the survival of patients. Pearson rank correlation was used to analyze the relationship between different genes or proteins.

References:

1. Wang L, Feng Z, Wang X, Wang X, Zhang X. DEGseq: an R package for identifying differentially expressed genes from RNA-seq data. Bioinformatics. 2010; 26: 136-8.

2. Robinson MD, McCarthy DJ, Smyth GK. edgeR: a Bioconductor package for differential expression analysis of digital gene expression data. Bioinformatics. 2010; 26: 139-40.

3. Li H, Durbin R. Fast and accurate short read alignment with Burrows-Wheeler transform. Bioinformatics. 2009; 25: 1754-60.

4. Li H, Handsaker B, Wysoker A, Fennell T, Ruan J, Homer N, et al. The Sequence Alignment/Map format and SAMtools. Bioinformatics. 2009; 25: 2078-9.

5. Boeva V, Popova T, Bleakley K, Chiche P, Cappo J, Schleiermacher G, et al. Control-FREEC: a tool for assessing copy number and allelic content using next-generation sequencing data. Bioinformatics. 2012; 28: 423-5.

6. Wang J, Mullighan CG, Easton J, Roberts S, Heatley SL, Ma J, et al. CREST maps somatic structural variation in cancer genomes with base-pair resolution. Nat Methods. 2011; 8: 652-4.

7. Wang K, Li M, Hakonarson H. ANNOVAR: functional annotation of genetic variants from high-throughput sequencing data. Nucleic Acids Res. 2010; 38: e164.

8. Cibulskis K, Lawrence MS, Carter SL, Sivachenko A, Jaffe D, Sougnez C, et al. Sensitive detection of somatic point mutations in impure and heterogeneous cancer samples. Nature biotechnology. 2013; 31: 213-9.

9. Saunders CT, Wong WS, Swamy S, Becq J, Murray LJ, Cheetham RK. Strelka: accurate somatic small-variant calling from sequenced tumor-normal sample pairs. Bioinformatics. 2012; 28: 1811-7.

10. Krueger F, Andrews SR. Bismark: a flexible aligner and methylation caller for Bisulfite-Seq applications. Bioinformatics. 2011; 27: 1571-2.

11. Langmead B, Salzberg SL. Fast gapped-read alignment with Bowtie 2. Nat Methods. 2012; 9: 357-9.

12. Lister R, Mukamel EA, Nery JR, Urich M, Puddifoot CA, Johnson ND, et al. Global epigenomic reconfiguration during mammalian brain development. Science. 2013; 341: 1237905.

13. Feng H, Conneely KN, Wu H. A Bayesian hierarchical model to detect differentially methylated loci from single nucleotide resolution sequencing data. Nucleic Acids Res. 2014; 42: e69.

14. Wu H, Xu T, Feng H, Chen L, Li B, Yao B, et al. Detection of differentially methylated regions from whole-genome bisulfite sequencing data without replicates. Nucleic Acids Res. 2015; 43: e141.

15. Park Y, Wu H. Differential methylation analysis for BS-seq data under general experimental design. Bioinformatics. 2016; 32: 1446-53.

16. Wang Y, Ding Q, Yen CJ, Xia W, Izzo JG, Lang JY, et al. The crosstalk of mTOR/S6K1 and Hedgehog pathways. Cancer Cell. 2012; 21: 374-87.

17. Huang da W, Sherman BT, Lempicki RA. Systematic and integrative analysis of large gene lists using DAVID bioinformatics resources. Nat Protoc. 2009; 4: 44-57.

18. Subramanian A, Tamayo P, Mootha VK, Mukherjee S, Ebert BL, Gillette MA, et al. Gene set enrichment analysis: a knowledge-based approach for interpreting genome-wide expression profiles. Proc Natl Acad Sci U S A. 2005; 102: 15545-50.
